# Palmitoylation‐Mediated Ubiquitination of SRPK1 Regulates Ferroptosis in High‐Fat‐Induced Erectile Dysfunction

**DOI:** 10.1002/advs.202513796

**Published:** 2026-01-29

**Authors:** Xiao‐Hui Tan, Ke‐Fan Li, Yi‐Ming Yuan, Man‐Cheng Xia, Fang‐Zhou Zhao, Hong‐Gang Ying, Zhuo Zhou, Peng‐Chao Gao, Guo‐Qing Xie, Xue‐Song Li, Hui Jiang, Rui‐Li Guan

**Affiliations:** ^1^ Department of Urology Peking University First Hospital Beijing P. R. China; ^2^ Institute of Urology Peking University Beijing P. R. China; ^3^ National Urological Cancer Center Beijing P. R. China

**Keywords:** endothelial cells, erectile dysfunction, p53, S‐palmitoylation, SRPK1

## Abstract

Serine‐arginine protein kinase 1 (SRPK1) is a major protein kinase involved in mRNA splicing, cell cycle, and endothelial function. Recent studies have highlighted a close relationship between palmitic acid (PA) and endothelial cell ferroptosis. Here, we demonstrate that PA promotes the ubiquitination‐dependent degradation of SRPK1 mediated by the E3 ubiquitin ligase mindbomb 1 (MIB1) at lysine 494. Moreover, SRPK1 S‐palmitoylation is catalyzed by zinc‐finger DHHC S‐acyltransferase 24 (ZDHHC24) at cysteines 188/502/647 and deacylated by acyl protein thioesterase 1 (APT1). The dynamic S‐palmitoylation of SRPK1 can strengthen SRPK1‐MIB1 interaction, facilitate its ubiquitination, and thereby affect protein stability. Furthermore, SRPK1 modulates the phosphorylation of p53 tumor suppressor protein (p53) at serine 15, which may promote its nuclear translocation and activation under PA stimulation or in high‐fat‐diet‐fed animal models. The crucial effect of SRPK1 on p53 activation contributes to the suppression of endothelial cell ferroptosis in the context of lipid accumulation. Additionally, in silico screening reveals that 4'‐O‐Methylochnaflavone interacts with SRPK1, which effectively stabilizes SRPK1 and alleviates PA‐induced ferroptosis. Collectively, these findings underscore the critical role of PA in regulating endothelial cell ferroptosis via SRPK1 S‐palmitoylation and p53 activation, providing potential therapeutic strategies for dyslipidemia‐related erectile dysfunction.

## Graphical abstract

Elevated exogenous palmitic acid promotes the S‐palmitoylation of SRPK1 in endothelial cells, a dynamic process governed by ZDHHC24 and APT1. This post‐translational modification strengthens the interaction between SRPK1 and the E3 ubiquitin ligase MIB1, thereby facilitating the proteasomal degradation of SRPK1. The resulting loss of SRPK1 leads to diminished p53 phosphorylation at Ser15, which compromises its stability. Ultimately, this endothelial SRPK1 deficiency triggers GPX4‐dependent ferroptosis, contributing to the pathogenesis of high‐fat‐induced erectile dysfunction.

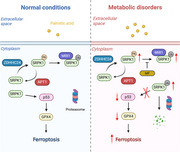

## 1 Introduction

Erectile dysfunction (ED), defined as the persistent inability to achieve or maintain an erection sufficient for satisfactory sexual activity, significantly impairs quality of life and psychosocial well‐being [1, 2]. Although oral phosphodiesterase inhibitors remain the mainstay of treatment, ongoing basic and translational research continues to uncover novel pathophysiological mechanisms and therapeutic targets. Importantly, ED also serves as an early clinical marker for major cardiovascular diseases, offering a valuable tool for identifying men at elevated cardiovascular risk [3]. Common comorbidities, including hypertension, obesity, dyslipidemia, and diabetes mellitus, underscore its metabolic and vascular underpinnings in the development of ED. Notably, hyperlipidemia is present in up to 42.4% of men with ED, and abnormal cholesterol levels are significantly correlated with moderate‐to‐severe disease [1, 4]. Despite this high prevalence, hyperlipidemia‐related ED remains relatively underexplored at the mechanistic level compared with diabetes mellitus‐induced or neurogenic‐associated ED. Deeper understandings of the cellular and molecular mechanisms of ED caused by a high‐fat diet (HFD), particularly in the context of vascular health, are therefore essential for identifying novel therapeutic targets and developing effective interventions.

The formation of a functional vascular network is essential to cellular health throughout the whole body, particularly within the cardiovascular system [5]. Endothelial cells, which constitute the inner surface of blood and lymphatic vessels, play pivotal roles in nutrient and oxygen delivery, immune cell trafficking, blood flow regulation, and maintenance of tissue homeostasis [6]. Dysfunction and death of these cells are closely associated with chronic diseases such as diabetes mellitus, hypertension, dyslipidemia, and obesity. Among various metabolic alterations, palmitic acid (PA) has a profound impact on vascular function, immunity, and inflammation. As a prevalent saturated fatty acid found in palm oil and animal fat, PA has been extensively documented as a major fatty acid substrate contributing to protein S‐palmitoylation [7, 8, 9]. Protein palmitoylation, also referred to as S‐acylation or S‐palmitoylation, is a reversible lipid modification based on a thioester linkage between PA and the cysteine residue, which can influence subcellular localization associated with membrane domains, protein‐protein interactions, and protein stability [10]. Previous studies have suggested an association between PA exposure and the progression of endothelial cell dysfunction and ferroptosis, implicating its role in the pathogenesis of cardiovascular diseases and ED that share common risk factors [3, 11, 12]. Understanding the mechanisms underlying regulated endothelial cell death may broaden the therapeutic strategies in patients with obesity and vascular complications.

Serine‐arginine protein kinases (SRPKs) represent a distinct subfamily of protein kinases that selectively phosphorylate splicing factors enriched in serine/arginine (SR) domains, playing a pivotal role in mRNA splicing, tumorigenesis, and endothelial function [12, 13, 14]. In particular, SRPK1 regulates angiogenesis by modulating the alternative splicing of vascular endothelial growth factor A (VEGF‐A), promoting the expression of pro‐angiogenic isoforms and thereby influencing new blood vessel formation [15]. Moreover, SRPK1 can function as both an oncogene and a tumor suppressor by regulating Akt activation through its interaction with the Akt phosphatase PHLPP1 [16]. Previous research has emphasized the crucial role of the SRPK1/Akt axis in PA‐induced endothelial cell dysfunction and impaired cell viability [12]. The enzymatic activity of SRPK1 is fine‐tuned by multiple mechanisms, including autophosphorylation. Another layer of regulation is provided by TIP60‐dependent acetylation, which modulates its autophosphorylation, stability, and cellular distribution [17]. It has been proposed that cyclin D1 up‐regulation triggers cell cycle progression in the nervous system, in which SRPK2 phosphorylates and activates the splicing factor SC35 and then impairs the phosphorylation at serine (Ser, S) 15 of p53 protein [18, 19]. However, the role of SRPK1 in the p53 signaling pathway and its other post‐translational modifications (PTMs) remain elusive.

In this study, we reveal that ZDHHC24‐mediated S‐palmitoylation of SRPK1, a process counteracted by APT1, is associated with enhanced ubiquitination and proteasomal degradation. This regulatory mechanism links SRPK1 to p53 activation and endothelial cell ferroptosis in response to PA and HFD, highlighting SRPK1 as a potential therapeutic target for dyslipidemia‐related ED and vascular dysfunction.

## 2 Results

### 2.1 PA Facilitates the Ubiquitination‐Dependent Degradation of SRPK1

**FIGURE 1:**
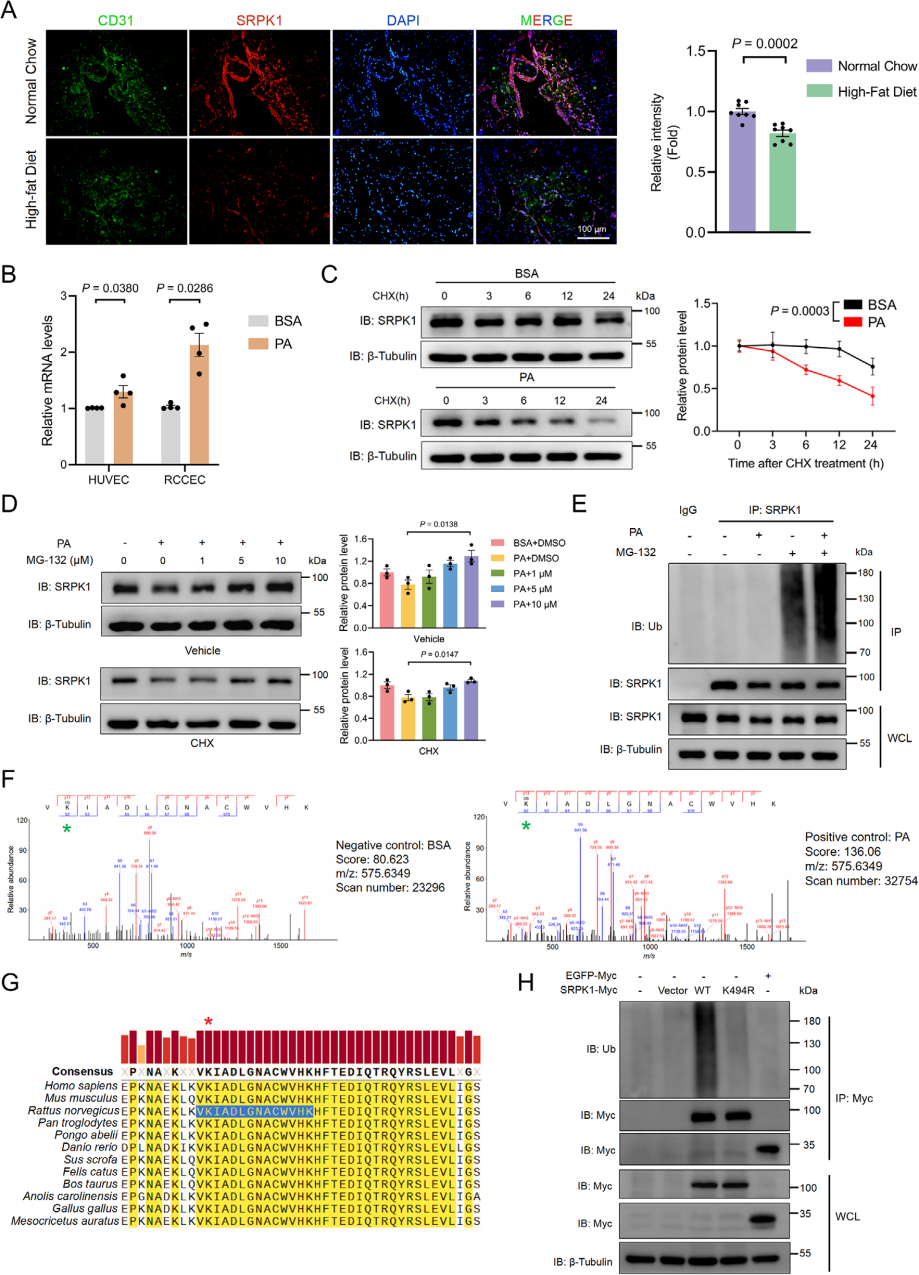
PA facilitates the ubiquitination‐dependent degradation of SRPK1 (A). Immunofluorescence (left) and semi‐quantification (right, n = 8 rats/group) were performed to analyze the expression of SRPK1 (red) in endothelial cells marked by CD31 (green) on corpus cavernosum sections of rats fed with normal chow or high‐fat diet. Scale bars, 100 µm. Nuclei, DAPI, blue. (B) Relative mRNA levels of SRPK1 in endothelial cells treated with BSA or PA (0.25 mm). Data represent the mean ± s.e.m. of four independent experiments. *P* values were calculated using a two‐sided unpaired t‐test or non‐parametric tests as appropriate (C). The stability of SRPK1 in RCCEC in the presence of BSA or PA (0.25 mm) was assessed at different time points after the treatment of cycloheximide (CHX, 50 µg/mL), an inhibitor of protein biosynthesis. Data represent the mean ± s.e.m. of six independent experiments. *P* values were calculated by two‐way ANOVA analysis (D). RCCEC were treated with PA (0.25 mm) and MG‐132, a potent and reversible proteasome inhibitor, at the indicated concentrations (0–10 µm) in the presence of vehicle or cycloheximide (CHX, 50 µg/mL). Data represent the mean ± s.e.m. of three independent experiments. *P* values were calculated by one‐way ANOVA followed by Tukey's multiple comparison test (E). RCCEC were treated with PA (0.25 mm) and MG‐132 (10 µm) as indicated. Lysates were immunoprecipitated with anti‐SRPK1, and immunoblot analysis was performed to analyze the levels of ubiquitination. IP, immunoprecipitation; WCL, whole‐cell lysates (F). The ubiquitination of lysine (K) was identified using LC‐MS analysis. The potential modifications of V(K)IADLGNACWVHK were shown, and the ubiquitination site was marked with a green asterisk (G). Sequence conservation of the ubiquitinated sequence (blue) and site (red asterisk) of SRPK1 across different species. The consensus with a threshold of more than 95% was highlighted (yellow), with the sequence conservation shown in colored bars (H). HEK293T cells transfected with the indicated DNA constructs were treated with MG‐132 (10 µm, 6 h). Lysates were immunoprecipitated with anti‐Myc to analyze the levels of ubiquitination through immunoblot analysis.

To investigate the involvement of SRPK1 in HFD‐induced endothelial dysfunction and cell death, we first measured its expression in the corpus cavernosum of rats fed with either normal chow or HFD using immunofluorescence (IF). The results showed that SRPK1 protein levels were significantly reduced in endothelial cells from HFD‐fed rats compared to controls (Figure 1A), consistent with our previous observation that PA exposure decreased endothelial SRPK1 expression in a concentration‐dependent manner [12].

To elucidate the mechanism behind the reduced protein levels, we examined SRPK1 at both the transcriptional and post‐translational levels. Notably, mRNA levels of *SRPK1* were elevated in PA‐treated human umbilical vein endothelial cells (HUVEC) and rat corpus cavernosum endothelial cells (RCCEC), suggesting that its transcription was not impaired in these settings (Figure 1B). Cycloheximide (CHX) chase assays revealed a reduced half‐life of endogenous SRPK1 in RCCEC treated with PA, indicating that PA markedly compromises SRPK1 protein stability (Figure 1C). Further investigation showed that the proteasome inhibitor MG‐132 effectively blocked PA‐induced SRPK1 degradation, whereas the autophagy inhibitor hydroxychloroquine (HCQ) had no significant effect (Figure 1D; Figure S1A). Consistently, immunoblotting with an anti‐ubiquitin antibody revealed marked polyubiquitination of SRPK1 in PA‐treated RCCEC (Figure 1E). These findings implicate the role of the ubiquitin‐proteasome system (UPS) in the degradation process of SRPK1.

To explore lysine residues potentially involved in SRPK1 polyubiquitination, we performed a ubiquitinated proteome mass spectrometry analysis in endothelial cells treated with BSA or PA. Notably, a ubiquitinated peptide encompassing lysine 494 (K494) of human SRPK1, a residue evolutionarily conserved from fish to mammals, was significantly enriched in PA‐treated cells compared to controls (Figure 1F,G). Accordingly, we generated a lysine‐to‐arginine (K494R) mutant in the kinase domain. Exogenously expressed SRPK1‐K494R‐Myc showed significantly reduced polyubiquitination compared with the wild type (SRPK1‐WT‐Myc) (Figure 1H). Intriguingly, although the lysine‐containing peptide VKIADLGNACWVHK is also present in SRPK2, PA treatment increased SRPK2 protein levels in a dose‐ and time‐dependent manner without a corresponding increase in polyubiquitination levels (Figure S1B–E).

### 2.2 E3 Ubiquitin Ligase MIB1 Regulates Ubiquitination Degradation of SRPK1

**FIGURE 2:**
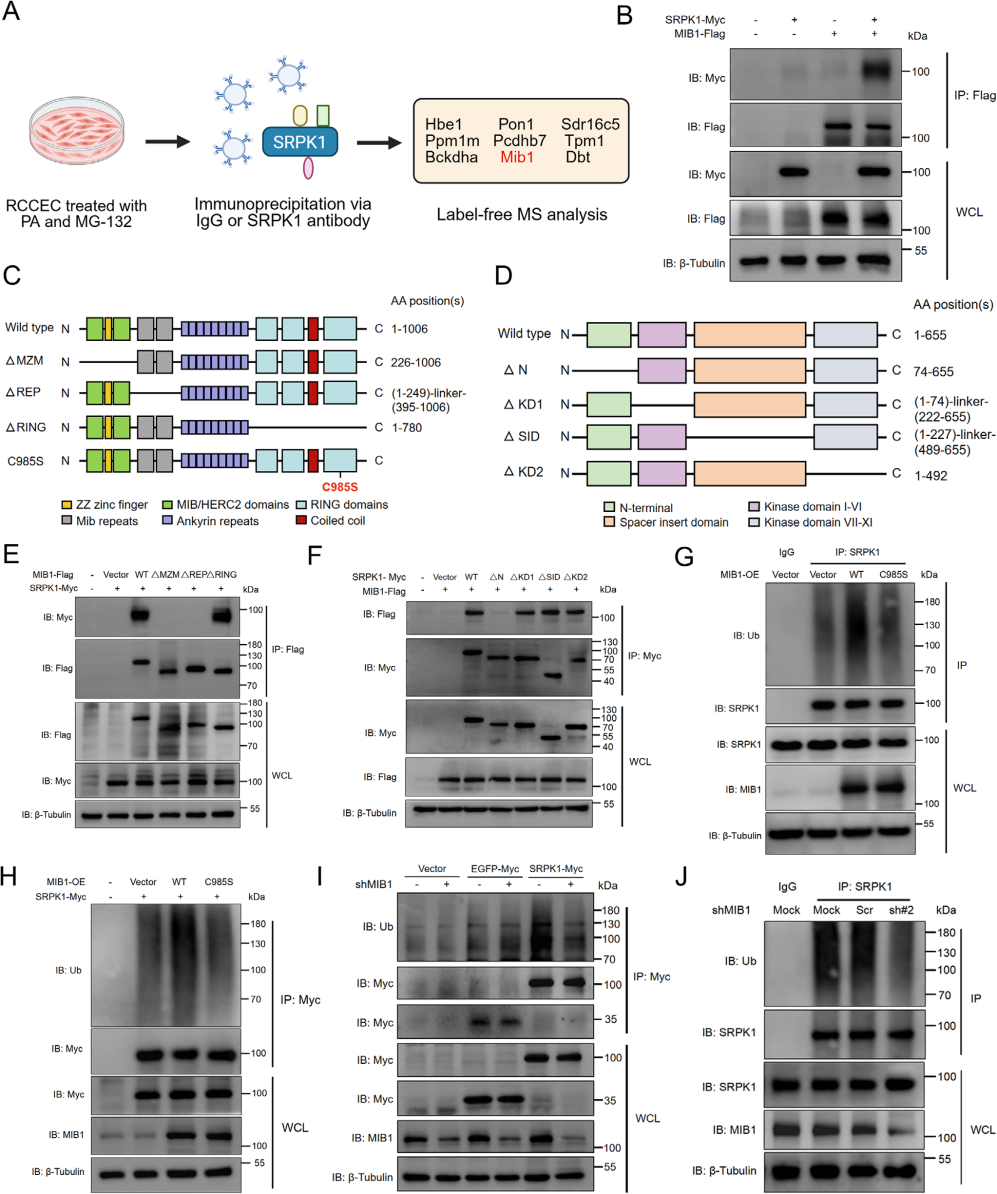
E3 ubiquitin ligase MIB1 regulates ubiquitination‐dependent degradation of SRPK1 (A). Schematic workflow of immunoprecipitation (IP) using an anti‐SRPK1 antibody or an IgG control. The top‐ranked genes enriched in the anti‐SRPK1 group are highlighted in box (B). Lysates from HEK293T cells transfected with DNA constructs as indicated and treated with MG‐132 (10 µm, 6 h) were immunoprecipitated with anti‐Flag. Immunoblot analysis was performed to analyze the interaction between MIB1‐Flag and SRPK1‐Myc (C). MIB1's domain organization includes an MZM (short for Mib Herc2/ZZ Zinc finger/Mib‐Herc2) domain, a REP domain (two imperfect tandem repeats), an ANK domain, and a C‐terminal RING domain (D). Domain organization of SRPK1 contains an N‐terminal domain, a spacer insert domain, and two kinase domains (E). Lysates from HEK293T cells transfected with DNA constructs as indicated and treated with MG‐132 (10 µm, 6 h) were immunoprecipitated with anti‐Flag. Immunoblot analysis was performed to detect the presence of the indicated proteins (F). Lysates from HEK293T cells transfected with DNA constructs as indicated and treated with MG‐132 (10 µm, 6 h) were immunoprecipitated with anti‐Myc. Immunoblot analysis was performed to analyze the presence of indicated proteins (G). HEK293T cells transfected with MIB1‐WT or MIB1‐C985S were treated with MG‐132 (10 µm, 6 h). Lysates were immunoprecipitated with anti‐SRPK1, and western blots were performed to analyze the levels of ubiquitination (H). HEK293T cells were transfected with DNA constructs as indicated, then treated with MG‐132 (10 µm, 6 h). Lysates were immunoprecipitated with anti‐Myc, and immunoblot analysis was performed to analyze the levels of ubiquitination (I). HEK293T cells with knockdown of MIB1 were transfected with DNA constructs as indicated, then treated with MG‐132 (10 µm, 6 h). Lysates were immunoprecipitated with anti‐Myc, and immunoblot analysis was performed to analyze the levels of ubiquitination (J). HEK293T cells were transfected with scrambled shRNA (Scr) or MIB1 shRNA (shMIB1 #2) and treated with MG‐132 (10 µm, 6 h). Lysates were immunoprecipitated with anti‐SRPK1, and western blots were performed to analyze the levels of ubiquitination.

To identify the E3 ubiquitin ligase responsible for the polyubiquitination of SRPK1, we performed immunoprecipitation (IP) using an anti‐SRPK1 antibody or an IgG control incubated with lysates from RCCEC treated with PA and MG‐132, followed by mass spectrometry analysis (Figure 2A; Table S1). Notably, E3 ubiquitin ligase MIB1 was identified as the top‐ranked protein. MIB1 mediates ubiquitination of Delta receptors, which act as ligands of Notch proteins and induce a Notch signaling response [20]. Previous research has identified SRPK1 as a strong potential candidate for direct ubiquitination mediated by MIB1 using tandem ubiquitin binding entities assays [21]. Their interaction was confirmed by co‐immunoprecipitation (Co‐IP) assays in HEK293T cells co‐transfected with SRPK1‐Myc and MIB1‐Flag, which was further validated in RCCEC for endogenous SRPK1 and MIB1 (Figure 2B; Figure S2A).

The domain architecture of MIB1 comprises MZM, REP, ANK, and RING domains. The N‐terminal MZM and REP domains facilitate substrate recognition, while the RING domains mediate ubiquitin transfer via E2 conjugation (Figure 2C). On the other hand, SRPK1 constitutes an N‐terminal domain, a spacer insert domain, and two kinase domains (Figure 2D). We showed that MZM‐depleted and REP‐depleted MIB1 mutants abolished binding with SRPK1‐Myc compared with MIB1‐WT, indicating that MZM‐REP domains are required for binding to SRPK1 (Figure 2E). Meanwhile, deletion of the N‐terminal domain in SRPK1‐Myc significantly impaired its interaction with MIB1‐Flag, which implies that this region mediates its interaction with MIB1 (Figure 2F).

The protein levels of SRPK1 were decreased in cells with MIB1‐WT overexpression, whereas the catalytically inactive mutant MIB1‐C985S showed no significant effect (Figure S2B,C). Further ubiquitination assays demonstrated that MIB1‐WT enhanced SRPK1 polyubiquitination while MIB1‐C985S did not (Figure 2G,H). Conversely, knockdown of MIB1 by small hairpin RNA (shRNA; shMIB1) markedly restored SRPK1 protein stability and reduced its polyubiquitination in PA‐treated RCCEC (Figure S2D,E). Similarly, we also observed that knockdown of MIB1 in HEK293T cells affected the polyubiquitination levels of SRPK1 (Figure 2I,J). Taken together, these findings establish MIB1 as a key regulator of SRPK1 polyubiquitination and proteasomal degradation.

### 2.3 S‐Palmitoylation of SRPK1 at Cys188/502/647 is Essential for Its Degradation

**FIGURE 3:**
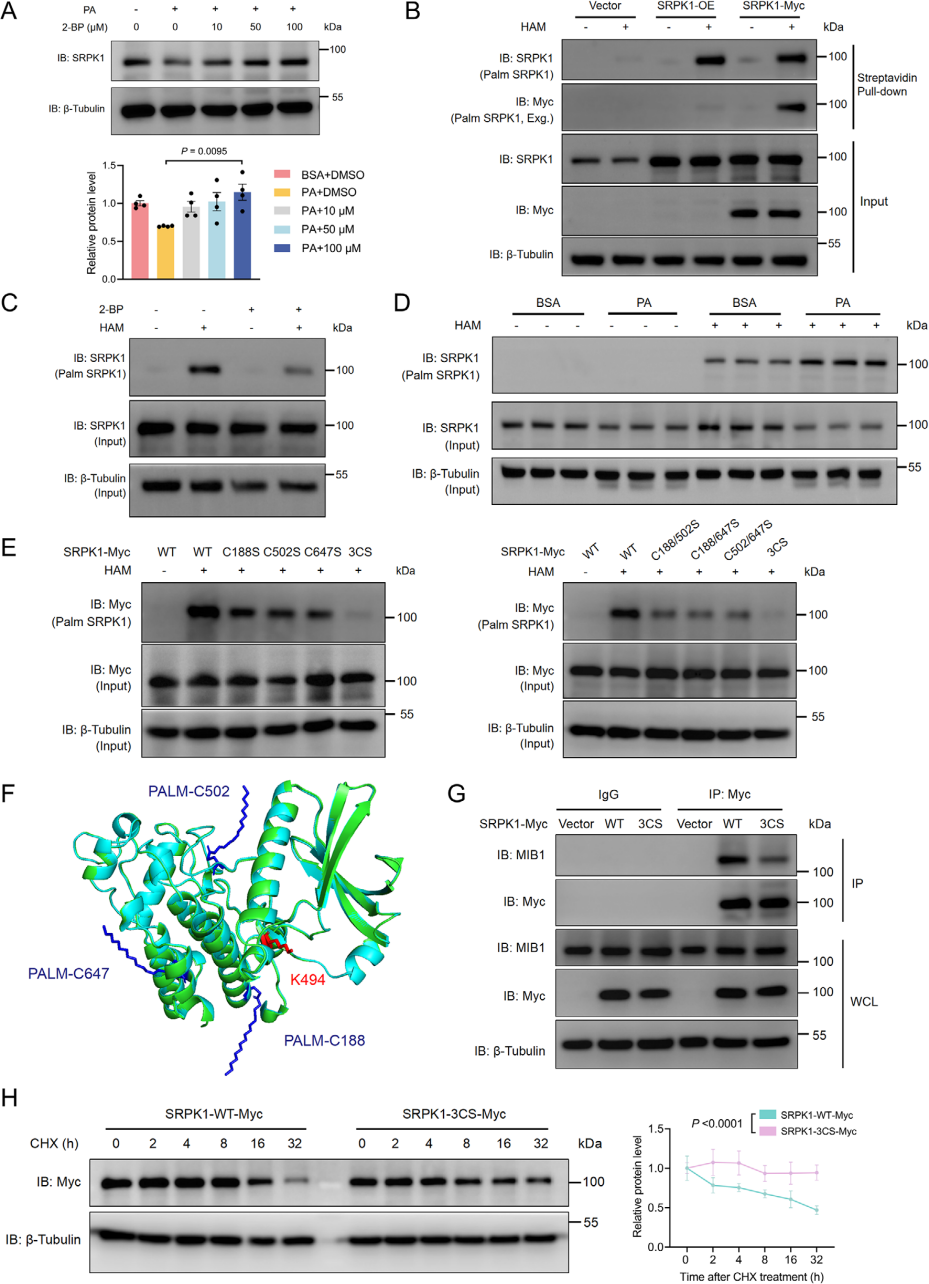
S‐palmitoylation of SRPK1 at Cys188/502/647 is essential for its degradation (A). RCCEC were treated with PA (0.25 mm) and 2‐bromopalmitate (2‐BP), a common and nonselective palmitoylation inhibitor, at the indicated concentrations (0–100 µm). Data represent the mean ± s.e.m. of four independent experiments. *P* values were calculated by one‐way ANOVA followed by Tukey's multiple comparison test (B). Acyl‐biotin exchange (ABE) assay and immunoblot analysis were performed to detect SRPK1 palmitoylation in HEK293T cells transfected with DNA constructs as indicated. HAM, hydroxylamine; Exg, Exogenous (C). ABE assay and immunoblot analysis of SRPK1 palmitoylation in RCCEC treated with 2‐BP (20 µm) (D). ABE assay and immunoblot analysis of SRPK1 palmitoylation in RCCEC treated with BSA or PA (0.25 mm) as indicated (n = 3 biological replicates) (E). ABE assay and immunoblot analysis showing SRPK1 palmitoylation in HEK293T cells transfected with SRPK1‐WT or indicated mutants with deficient predicted palmitoylation sites (F). AlphaFold structure prediction of core kinase domains of unmodified SRPK1 (cyans) and palmitoylated SRPK1 (green) at C188/502/647. The palmitoyl group (blue) covalently bound to C188/502/647 is shown in sticks, and so is the ubiquitination site K494 (red) (G). Lysates from HEK293T cells transfected with DNA constructs as indicated were immunoprecipitated with anti‐Myc. Immunoblot analysis was performed to analyze the interaction between MIB1 and SRPK1 mutants (H). The stability of SRPK1‐WT‐Myc and SRPK1‐3CS‐Myc in HEK293T cells was assessed at different time points after the treatment of cycloheximide (CHX, 100 µg/mL). Data represent the mean ± s.e.m. of six independent experiments. *P* values were calculated by two‐way ANOVA analysis.

PTMs are of great significance for protein function, localization, and stability, especially the sophisticated crosstalk among different PTMs [22]. Apart from ubiquitination, we hypothesized that S‐palmitoylation might contribute to the degradation of SRPK1, given that PA exposure can influence protein S‐palmitoylation dynamics. To experimentally validate the S‐palmitoylation of SRPK1, we treated RCCEC with 2‐bromopalmitate (2‐BP), a broad‐spectrum inhibitor of ZDHHC protein acyltransferases, which effectively rescued PA‐induced SRPK1 degradation (Figure 3A). In HEK293T cells, treatment with 2‐BP reduced the polyubiquitination levels of exogenously expressed SRPK1‐Myc without interfering with MIB1 expression (Figure S3A). In addition, we performed an acyl‐biotin exchange (ABE) assay in HEK293T cells overexpressing SRPK1 to selectively detect palmitoylated proteins. Free thiol groups were first blocked with N‐ethylmaleimide (NEM), followed by hydroxylamine (HAM)‐mediated cleavage of palmitoyl modifications and subsequent biotinylation. Biotinylated proteins were then enriched using streptavidin magnetic beads and analyzed by immunoblotting (Figure 3B). Our results demonstrated that SRPK1 S‐palmitoylation levels were reduced in RCCEC treated with 2‐BP but enhanced upon PA exposure (Figure 3C,D). These findings indicated that SRPK1 undergoes S‐palmitoylation that may influence its stability.

To identify the key palmitoylation sites in SRPK1, we used a S‐palmitoylation site prediction tool (GPS‐Palm software) to filter the possibly palmitoylated sites of SRPK1 [23]. We found that cysteines (Cys, C) 188, 502, and 647 of human SRPK1 were conserved across species with a relatively high score (Figure S3B,C). To validate these sites, palmitoylation‐deficient mutants (cysteine to serine) were generated and tested using ABE assays, revealing that mutation of either one or two of C188/C502/C647 partially affected SRPK1 S‐palmitoylation and that mutating three residues simultaneously (3CS) abolished SRPK1 S‐palmitoylation (Figure 3E). Additionally, polyubiquitination levels of SRPK1 were significantly decreased in the 3CS mutant, suggesting that S‐palmitoylation at these sites is essential for SRPK1 polyubiquitination (Figure S3D).

To gain insights into the structural impact of S‐palmitoylation on SRPK1 degradation, we performed in silico structural modeling using AlphaFold. The predicted global structure of SRPK1 with palmitoylation at C188/C502/C647 closely resembled that of the unmodified protein, suggesting that S‐palmitoylation does not induce large‐scale conformational changes (Figure 3F; Figure S3E,F). As three palmitoylated residues are spatially proximal to the ubiquitination site K494 and S‐palmitoylation can regulate protein‐protein interaction, we hypothesize that S‐palmitoylation may influence the interaction between MIB1 and SRPK1, thereby affecting SRPK1 degradation. Co‐IP assays confirmed that the binding between MIB1 and SRPK1‐WT was strengthened compared to the SRPK1‐3CS mutant, suggesting that SRPK1 S‐palmitoylation promotes SRPK1‐MIB1 interaction (Figure 3G). Subsequent CHX chase assays demonstrated that the half‐life of SRPK1‐3CS was extended compared with SRPK1‐WT, revealing that SRPK1 S‐palmitoylation at C188/C502/C647 promotes protein degradation (Figure 3H). Collectively, these data support that S‐palmitoylation of SRPK1 at Cys188/502/647 is essential for MIB1‐mediated ubiquitination and proteasomal degradation.

### 2.4 APT1 and ZDHHC24 Mediates S‐Palmitoylation of SRPK1

**FIGURE 4:**
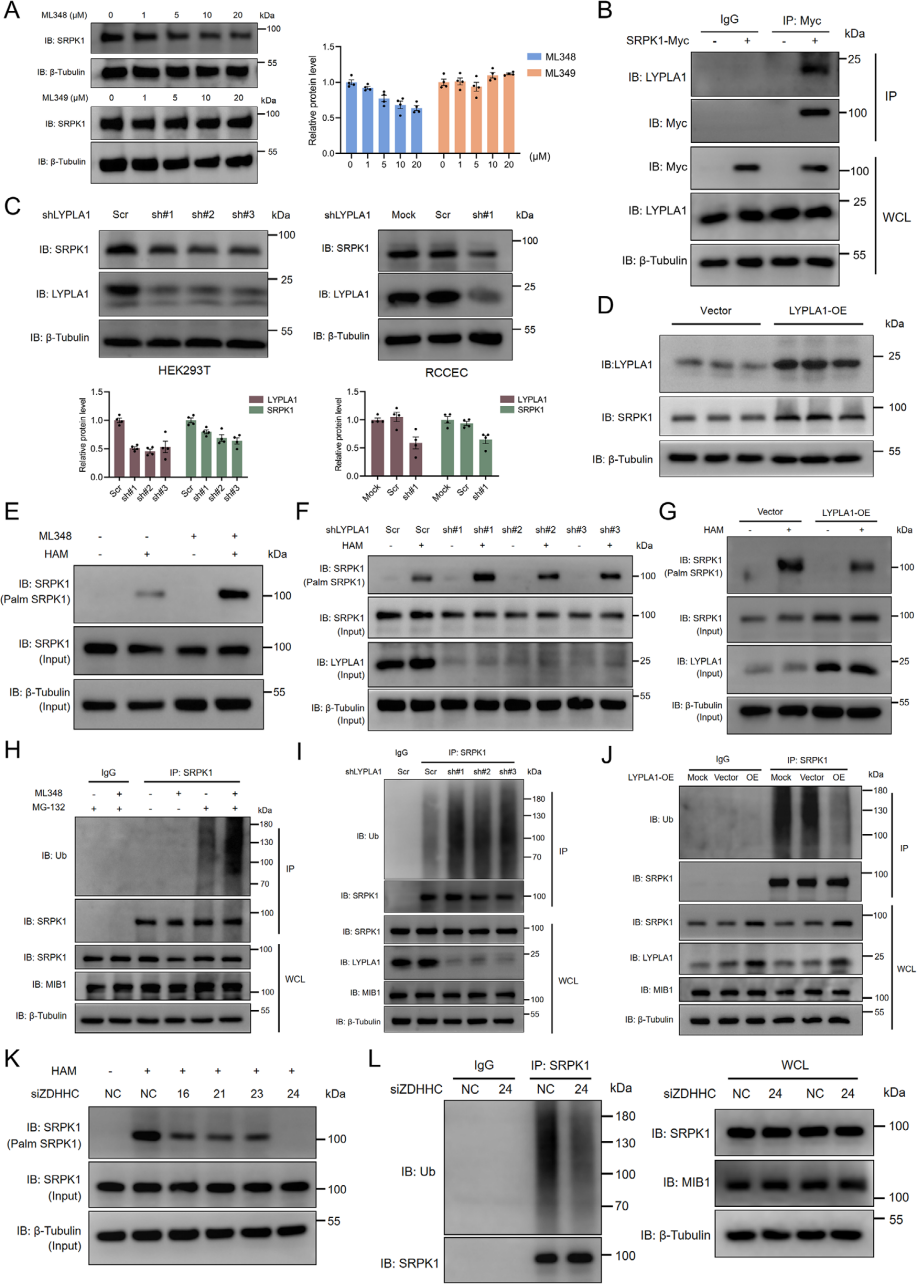
APT1 and ZDHHC24 mediate S‐palmitoylation of SRPK1 (A). RCCEC were treated with ML348, a selective and reversible inhibitor of acyl protein thioesterase 1 (APT1, also known as lysophospholipase 1 encoded by gene *LYPLA1*), and ML349, a potent and specific acyl protein thioesterase 2 (APT2) inhibitor, at the indicated concentrations (0‐20 µm). Data represent the mean ± s.e.m. of four independent experiments (B). Lysates from HEK293T cells transfected with DNA constructs as indicated were immunoprecipitated with anti‐Myc. Immunoblot analysis was performed to analyze the interaction between LYPLA1 and SRPK1 (C). RCCEC and HEK293T cells were transfected with scrambled shRNA (Scr) or LYPLA1 shRNAs (shLYPLA1 #1, shLYPLA1 #2, shLYPLA1 #3) as indicated. The protein levels of SRPK1 and LYPLA1 were analyzed using immunoblot analysis. Data represent the mean ± s.e.m. of four independent experiments (D). HEK293T cells were transfected with the indicated DNA constructs for 72 h. The protein levels of SRPK1 and LYPLA1 were analyzed by immunoblot analysis (n = 3 biological replicates) (E). ABE assay and immunoblot analysis of SRPK1 palmitoylation in HEK293T cells treated with ML348. ABE assay, acyl‐biotin exchange assay; HAM, hydroxylamine (F). ABE assay and immunoblot analysis of SRPK1 palmitoylation in HEK293T cells with knockdown of LYPLA1 (G). ABE assay and immunoblot analysis of SRPK1 palmitoylation in HEK293T cells with overexpression of LYPLA1 (H). HEK293T cells were treated with ML348 and MG‐132 as indicated. Lysates were immunoprecipitated with anti‐SRPK1, and immunoblot analysis was performed to analyze the levels of ubiquitination (I). HEK293T cells with knockdown of LYPLA1 were treated with MG‐132. Lysates were immunoprecipitated with anti‐SRPK1, and immunoblot analysis was performed to analyze the levels of ubiquitination (J). HEK293T cells with overexpression of LYPLA1 were treated with MG‐132. Lysates were immunoprecipitated with anti‐SRPK1, and immunoblot analysis was performed to analyze the levels of ubiquitination (K). After transfection with the indicated siRNAs in HEK293T cells for 48 h, the cellular lysates were subjected to the ABE assay (L). HEK293T cells transfected with the indicated siRNAs were treated with MG‐132. Lysates were immunoprecipitated with anti‐SRPK1, and immunoblot analysis was performed to analyze the levels of ubiquitination.

Protein S‐palmitoylation is catalyzed by a family of zinc finger DHHC domain‐containing palmitoyltransferases (ZDHHCs), with 23 members identified in mammals, and is counteracted by depalmitoylases such as acyl protein thioesterases (APTs), the lysosomal hydrolase PPT1, and members of the ABHD protein family [10]. We observed that pharmacological inhibition of APT1 encoded by gene *LYPLA1* with ML348, but not inhibition of APT2 with ML349, resulted in a marked reduction of SRPK1 protein levels (Figure 4A; Figure S4A). This observation was corroborated by Co‐IP analysis, which demonstrated an interaction between SRPK1 and APT1 (Figure 4B). Moreover, both genetic knockdown and pharmacological inhibition of APT1 led to increased SRPK1 S‐palmitoylation and decreased protein levels, whereas APT1 overexpression elicited the opposite effect (Figure 4C–G; Figure S4B). In line with these findings, SRPK1 polyubiquitination was significantly enhanced upon APT1 knockdown or inhibition, and reduced by APT1 overexpression (Figure 4H–J).

To pinpoint the palmitoyl acyltransferase responsible for SRPK1 S‐palmitoylation, we silenced various ZDHHC enzymes using validated siRNAs in HEK293T cells (Table S2) [9]. ABE assays revealed that knockdown of ZDHHC24 markedly diminished SRPK1 S‐palmitoylation compared with other ZDHHCs and reduced its polyubiquitination (Figure 4K,L; Figure S4C,D). We then confirmed that ZDHHC24 interacted with SRPK1, resulting in a reduction in SRPK1 protein abundance (Figure S4E,F). All these findings indicate that APT1 and ZDHHC24 are likely the principal enzymes mediating the dynamic and reversible S‐palmitoylation of SRPK1.

### 2.5 SRPK1 Mediates p53 Phosphorylation at Ser15

**FIGURE 5:**
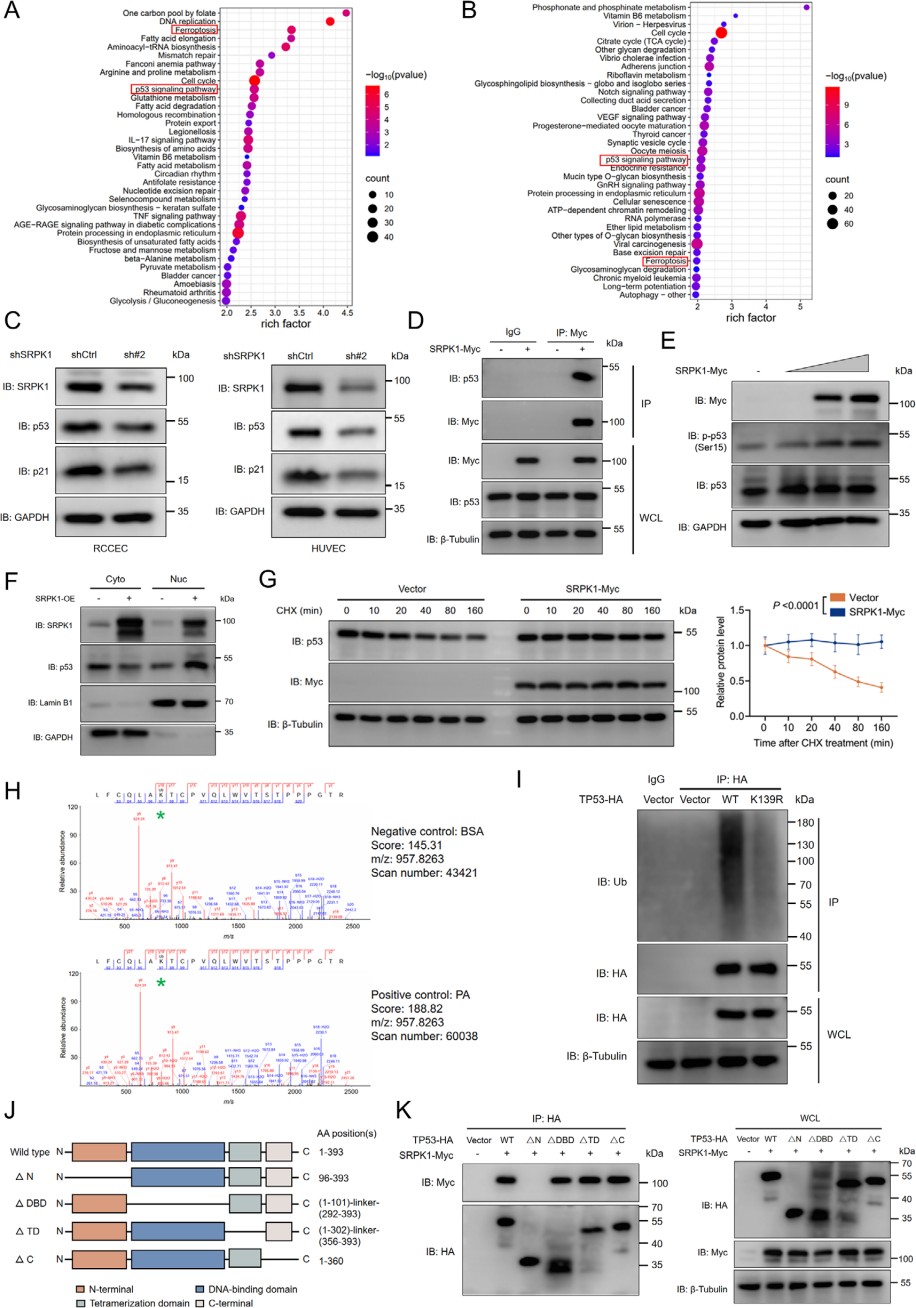
SRPK1 mediates p53 phosphorylation at Ser15 (A). The KEGG functional enrichment bubble plot was generated for the differentially expressed genes in RCCEC treated with PA or BSA (GEO: GSE205913). It depicted the top 35 most significantly altered pathways, including the p53 signaling pathway, ferroptosis, DNA replication, cell cycle, mismatch repair, and glutathione metabolism (B). KEGG functional enrichment bubble plot of differentially expressed genes in HEK293T cells with SRPK1 knockdown or scrambled shRNA (GEO: GSE283448), depicting the top 35 most significantly altered pathways, such as p53 signaling pathway, ferroptosis, cell cycle, VEGF signaling pathway, and cellular senescence (C). Immunoblot analysis of p53 and p21 in HUVEC and RCCEC transfected with shSRPK1 or shCtrl (D). Lysates from HEK293T cells transfected with the indicated DNA constructs were immunoprecipitated with anti‐Myc. Immunoblot analysis was performed to analyze the interaction between p53 and SRPK1 (E). HEK293T cells were transfected with SRPK1‐Myc at doses of 0, 0.5, 1.5, and 4.5 µg. The protein levels of phosphorylated Ser15 and total p53 were assayed by immunoblot analysis (F). The nucleus/cytoplasm fractionation of p53 and SRPK1 in HEK293T cells transfected with the indicated DNA constructs (G). The stability of p53 in HEK293T cells transfected with SRPK1‐Myc or Vector was assessed at different time points after the treatment of cycloheximide (CHX, 100 µg/mL). Data represent the mean ± s.e.m. of six independent experiments. *P* values were calculated by two‐way ANOVA analysis (H). The ubiquitination of lysine (K) was identified using LC‐MS analysis. The potential modifications of LFCQLA(K)TCPVQLWVSTPPPGTR were shown, and the ubiquitination site was marked with a green asterisk (I). HEK293T cells were transfected with DNA constructs and treated with MG‐132 (10 µm, 6 h). Lysates were immunoprecipitated with anti‐HA, and immunoblot analysis was performed to analyze the levels of ubiquitination (J). Domain organization of p53 contains an N‐terminal domain, a DNA binding domain (DBD), a tetramerization domain (TD), and a C‐terminal domain (K). Lysates from HEK293T cells transfected with the indicated DNA constructs were immunoprecipitated with anti‐HA. Immunoblot analysis was performed to analyze the presence of the indicated proteins.

Our previous RNA sequencing (RNA‐seq) datasets (GEO: GSE205913) revealed significant enrichment of the p53 signaling pathway and ferroptosis in endothelial cells treated with PA, a condition that concurrently diminished SRPK1 protein levels (Figure 5A). To further delineate the role of SRPK1 in these pathways, we performed RNA‐seq on total RNA extracted from HEK293T cells following SRPK1 knockdown (GEO: GSE283448). Gene set enrichment analysis demonstrated significant changes of genes associated with p53 activity across pathways, including the p53 signaling pathway, ferroptosis, cell cycle regulation, and cellular senescence (Figure 5B). In addition, the VEGF signaling pathway emerged as one of the top‐ranked pathways, which is consistent with the well‐established link between VEGF alternative splicing and SRPK1 [15, 24]. Further analyses, including GSEA and examination of alternative splicing events, confirmed the loss of SRPK1 function (Figure S5A–C). We observed that SRPK1 knockdown reduced p53 protein levels in endothelial cells, whereas overexpression of SRPK1 abrogated this effect in PA‐treated RCCEC (Figure 5C; Figure S5D). Our mass spectrometry analysis initially suggested a potential interaction between SRPK1 and p53 (Table S1), which was subsequently confirmed by Co‐IP analysis (Figure 5D). Importantly, the mRNA levels of *TP53* remained largely unchanged in both RNA‐seq datasets (GEO: GSE205913, GSE283448), suggesting that SRPK1 possibly modulates p53 activity via PTMs rather than transcriptional regulation.

Although SRPK1 is traditionally recognized as a protein kinase for SR domain‐rich substrates involved in splicing, its potential role in regulating p53 phosphorylation has remained unclear. We found that SRPK1 promoted p53 phosphorylation at Ser15, which weakened its interaction with MDM2, reduced its polyubiquitination, and enhanced nuclear accumulation of p53 (Figure 5E,F; Figure S5E,F). Consistently, CHX chase assays revealed a prolonged half‐life of p53 in HEK293T cells overexpressing SRPK1, indicating that SRPK1 contributes to p53 protein stabilization (Figure 5G). Moreover, the disruption of SRPK1 S‐palmitoylation via APT1 may affect the phosphorylation of p53 on Ser15 (Figure S5H). In addition to SRPK1, ubiquitinated proteome mass spectrometry identified a ubiquitinated peptide encompassing lysine 139 (K139) of human p53 that was markedly increased in PA‐treated cells (Figure 5H; Figure S5G–I). Co‐IP and immunoblotting assays further demonstrated that the p53‐K139R‐HA mutant exhibited significantly reduced polyubiquitination compared to p53‐WT‐HA, possibly affecting the stability of p53 protein (Figure 5I). The domain organization of p53 consists of the N‐terminal domain (N), DNA‐binding domain (DBD), tetramerization domain (TD), and C‐terminal domain (C) (Figure 5J). We showed that depletion of the N‐terminal in p53 abolished binding with SRPK1‐Myc compared with wild types, indicating that N‐terminal domains bind to SRPK1 (Figure 5K). Collectively, these findings suggest that SRPK1 mediates p53 phosphorylation at Ser15.

### 2.6 SRPK1 Attenuates PA‐Associated Endothelial Cell Ferroptosis

**FIGURE 6:**
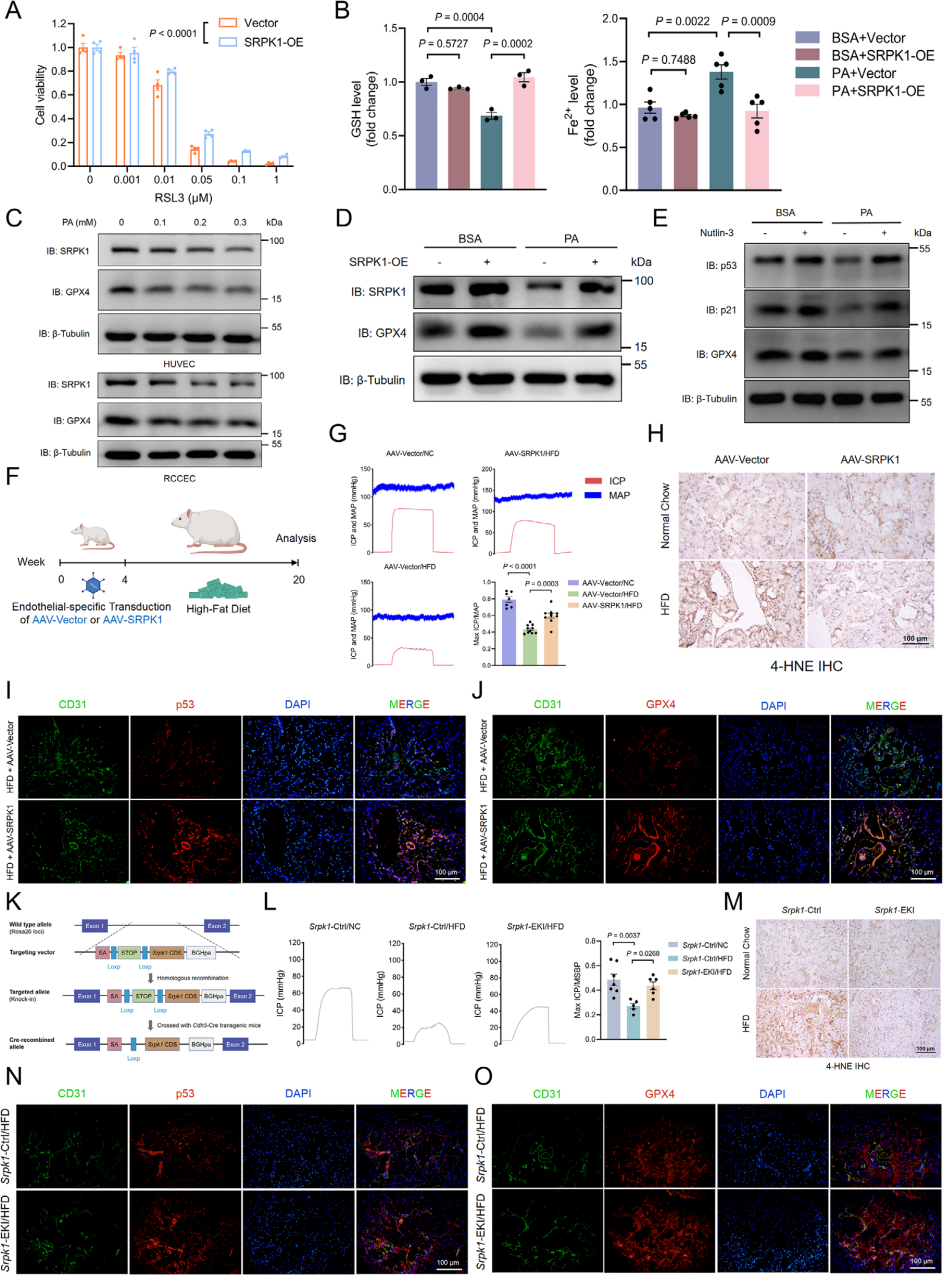
SRPK1 attenuates PA‐associated endothelial cell ferroptosis (A). Cell viability was measured after treating RCCEC overexpressing SRPK1 with RSL3, an inhibitor of glutathione peroxidase 4 (GPX4), for 24 h. Data represent the mean ± s.e.m. of 4 wells of a 96‐well plate. *P* values were calculated by two‐way ANOVA analysis (B). The levels of GSH (left) and Fe^2+^ (right) in endothelial cells overexpressing SRPK1 were measured following PA (0.25 mm) stimulation or BSA (C). RCCEC and HUVEC were treated with PA at the indicated concentration, and whole‐cell lysate was analyzed for immunoblot detection of SRPK1 and GPX4 (D). Immunoblot showing SRPK1 and GPX4 expression in RCCEC overexpressing SRPK1 treated with BSA or PA (0.25 mm) (E). Immunoblot analysis of p53, p21, and GPX4 in RCCEC stimulated with PA (0.25 mm) and Nutlin‐3 (20 µm) (F). Schematic diagram depicting HFD‐fed rats injected with endothelial‐specific AAV‐Vector and AAV‐SRPK1. Animals were locally injected with the required AAV in the corpus cavernosum for a 4‐week transduction, followed by a 16‐week HFD or NC. HFD, high‐fat diet; NC, normal chow (G). Representative intracavernous pressure (ICP) responses and mean arterial pressure (MAP) of male rats in the indicated group (n = 6–10 rats/group). The ratio of peak ICP/MAP was calculated to evaluate the erectile function (H). Representative immunohistochemistry (IHC) images of 4‐HNE in the corpus cavernosum of rats from the indicated groups. Scale bars, 100 µm. 4‐HNE, 4‐Hydroxynonenal (I). Images of immunofluorescence staining of corpus cavernosum tissues from HFD‐fed rats for p53 (red) and CD31 (green). Scale bars, 100 µm. Nuclei, DAPI, blue (J). Images of immunofluorescence staining of corpus cavernosum tissues from HFD‐fed rats for GPX4 (red) and CD31 (green). Scale bars, 100 µm. Nuclei, DAPI, blue (K). Schematic diagram of *Srpk1* endothelial‐specific knock‐in (*Srpk1*‐EKI) strategy. *Srpk1*‐EKI mice in a C57BL/6J background were generated using CRISPR/Cas9 and Cre‐loxP recombination technology. Deletion of the STOP cassette at the *Rosa26* locus results in the additional expression of its coding sequences (CDS) responsible for producing the SRPK1 protein. *Srpk1* CDS is expressed as an independent transcript driven by the *Rosa26* promoter. SA, splicing acceptor; BGHpa: bovine growth hormone polyadenylation (L). Representative intracavernous pressure (ICP) responses of male mice in the indicated group (n = 5–8 mice/group). The ratio of peak ICP/MSBP was calculated to evaluate the erectile function (M). Representative immunohistochemistry (IHC) images of 4‐HNE in the corpus cavernosum of mice from the indicated groups. Scale bars, 100 µm. 4‐HNE, 4‐Hydroxynonenal (N). Images of immunofluorescence staining of corpus cavernosum tissues from HFD‐fed mice for p53 (red) and CD31 (green). Scale bars, 100 µm. Nuclei, DAPI, blue (O). Images of immunofluorescence staining of corpus cavernosum tissues from HFD‐fed mice for GPX4 (red) and CD31 (green). Scale bars, 100 µm. Nuclei, DAPI, blue.

As an independent pathway for suppressing tumor growth, p53 plays a crucial yet complex role in regulating ferroptosis that is tightly linked to metabolism and oxidative stress responses [25, 26]. Previous studies have demonstrated that p21, a common p53 downstream molecule, exerts anti‐ferroptotic effects by modulating GPX4 [27, 28]. Notably, SRPK1 overexpression specifically inhibited ferroptosis induced by RSL3 (a direct inhibitor of GPX4) instead of Erastin (an inhibitor of the cystine/glutamate antiporter system X_c_^−^), highlighting the interaction between SRPK1 and GPX4 rather than an upstream regulation of cystine metabolism (Figure 6A; Figure S6A). Interestingly, RNA‐seq datasets (GEO: GSE211557) from RCCEC treated with SPHINX31, a selective inhibitor of SRPK1 enzymatic activity, exhibited significant enrichment of gene signatures related to ferroptosis and ferrous iron (Fe^2+^) homeostasis, implying that SRPK1 plays a regulatory role in endothelial cell ferroptosis (Figure S6B,C). Meanwhile, PA treatment had little effect on the protein levels of other known ferroptosis regulators, including FSP1 and ACSL4 (Figure S6D). PA‐induced endothelial cell death was rescued by the ferroptosis inhibitor ferrostatin‐1 (Fer‐1) instead of inhibitors targeting other forms of cell death, thus confirming its specificity for ferroptosis (Figure S6E). In contrast, oleic acid, another common saturated fatty acid, had no significant impact on SRPK1 and GPX4 expression (Figure S6F). Additionally, FTH1 and FTL1, key regulators of iron homeostasis, were markedly reduced in the SRPK1 knockdown group and upregulated in the SRPK1‐overexpression group, further consolidating the link between ferroptosis and SRPK1 (Figure S6G). Compared to the control group, PA‐induced ferroptosis was ameliorated in the SRPK1‐overexpression group, as evidenced by increased GSH levels, mitigated ROS accumulation, and reduced Fe^2+^ levels (Figure 6B; Figure S6H,I). We also found that overexpression of SRPK1 and activation of p53 ameliorated the GPX4 expression in PA‐treated RCCEC (Figure 6C–E).

To further validate the in vivo relevance between endothelial SRPK1 expression and dyslipidemia‐related ED, we generated an SRPK1 expression vector using adeno‐associated virus (AAV) type 9 (AAV‐SRPK1) that was locally injected into the corpus cavernosum (Figure 6F). Immunofluorescence analysis confirmed endothelial‐specific expressions of SRPK1 following AAV injection (Figure S7A,B). Compared to AAV‐Vector controls, rats with endothelial SRPK1 overexpression exhibited partial restoration of erectile function, although no significant changes were observed in body weight or serum lipid profiles following HFD feeding (Figure 6G; Figure S7C,D). Consistently, endothelial SRPK1 expression significantly reduced ferroptosis markers, as indicated by a decrease in 4‐HNE (4‐Hydroxynonenal, a lipid peroxidation product) levels and an increase in p53 and GPX4 expressions (Figure 6H–J). On the other hand, inhibition or knockdown of SRPK1 promoted ROS accumulation, elevated intracellular Fe^2+^ levels, and reduced GSH levels, suggesting that SRPK1 may play a decisive regulatory role in endothelial cell ferroptosis (Figure S8A–H).

Next, we generated endothelial‐specific *Srpk1* knock‐in (*Srpk1‐*EKI) mice by crossing *Rosa26^LSL‐Srpk1^* mice with VE‐Cadherin‐Cre (*Cdh5*‐Cre+) transgenic mice and fed the mice with a 16‐week HFD to induce ED (Figure S7E–H). Consistent with endothelial‐specific AAV intervention, we observed improved erectile function and reduced levels of 4‐HNE in HFD‐fed *Srpk1‐*EKI compared with controls (Figure 6K–M). The endothelial expressions of p53 and GPX4 were also increased in the HFD‐fed *Srpk1‐*EKI group (Figure 6N,O). These findings highlight the role of SRPK1 in mitigating endothelial cell ferroptosis and restoring vascular function.

### 2.7 4'‐O‐Methylochnaflavone Interacts with SRPK1 and Maintains its Stability

**FIGURE 7:**
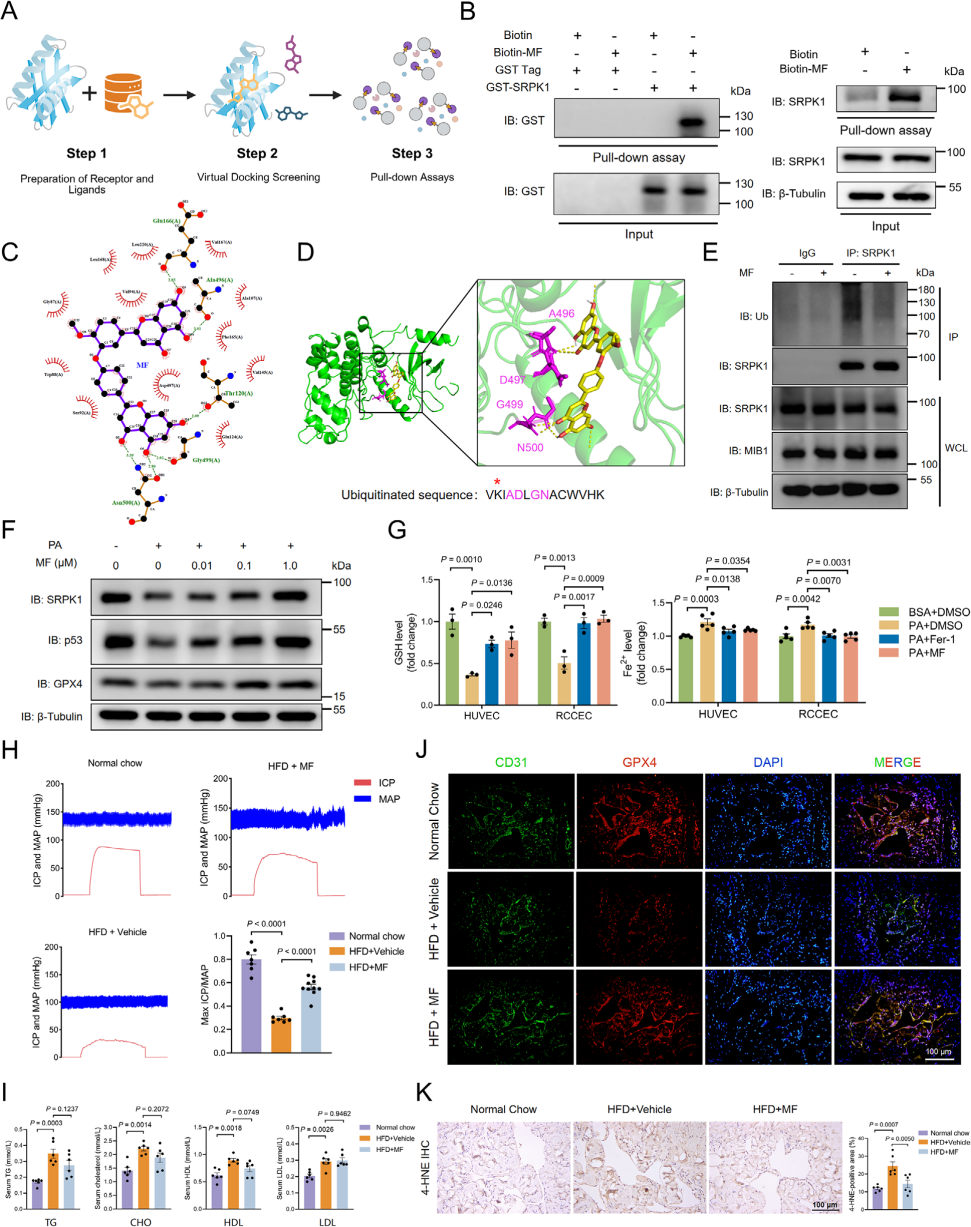
4'‐O‐Methylochnaflavone interacts with SRPK1 and maintains its stability (A). Schematic diagram illustrating the experimental workflow of the screening of small molecules that bind to SRPK1. A pocket located in SRPK1 was selected for targeted screening. Candidates were obtained by virtual docking screening followed by validations via pull‐down assays (B). Biotin‐MF pull‐down assays of recombinant SRPK1 protein (left) or endothelial cell lysates (right) after in vitro incubation overnight at 4°C. Representative immunoblot analysis of pull‐down assay from one of three independent experiments. Biotin alone was used as a control (C). Schematic diagram illustrating the binding of MF to the potential residues of SRPK1. The calculated binding free energy was −12.7 kcal/mol (D). Docking of MF (yellow) into the structure of SRPK1 (PDB ID: 5MY8, green) showing the interaction with predicted key residues (magenta) in the ubiquitinated sequence V(K)IADLGNACWVHK (E). RCCEC were treated with MF (1.0 µm) and MG‐132 (10 µm) as indicated. Lysates were immunoprecipitated with anti‐SRPK1, and immunoblot analysis was performed to analyze the levels of ubiquitination (F). Immunoblot assay of SRPK1, p53, and GPX4 in RCCEC treated with PA (0.25 mm) and MF at the indicated concentrations (0‐1.0 µm) (G). The levels of GSH (left) and Fe^2+^ (right) in endothelial cells treated with PA (0.25 mm) and MF (1.0 µm) or ferrostatin‐1 (Fer‐1, 20 µm) (H). Representative intracavernous pressure (ICP) responses and mean arterial pressure (MAP) of male rats in the indicated group (n = 6–10 rats/group). The ratio of peak ICP/MAP was calculated to evaluate the erectile function (I). Serum lipid profile of male rats from the indicated group, as determined by assay (n = 6–10 rats/group) (J). Immunofluorescence staining of corpus cavernosum tissues for GPX4 (red) and CD31 (green). Scale bars, 100 µm. Nuclei, DAPI, blue (K). Immunohistochemistry images of 4‐HNE in the corpus cavernosum of rats from the indicated groups (n = 6–10 rats/group). Scale bars, 100 µm.

Flavonoids are a class of polyphenolic compounds widely known for their potent antioxidant and anti‐inflammatory properties, playing a crucial role in maintaining vascular endothelial function by inhibiting oxidative stress and inflammation [29]. Our previous studies have demonstrated that icariside II could attenuate PA‐induced endothelial dysfunction via potential binding with SRPK1 and that hinokiflavone mitigated HFD‐induced ED via EGFR signaling [12, 30]. Based on this premise, small molecule compounds from a flavonoid library were screened for potential interactions with SRPK1 by in silico analysis. Affinity analysis revealed that 4'‐O‐Methylochnaflavone (MF) exhibits relatively high binding ability (Figure 7A; Table S3). As a positive control, SPHINX31 that occupied a binding pocket of SRPK1 showed a comparable binding affinity (Figure S9A and Table S3). In addition, we further confirmed the interaction between SRPK1 and MF via Biotin‐MF pull‐down (Figure 7B; Figure S9B,C). Given that the potential binding sites of MF in complex with SRPK1 were located near the polyubiquitination site K494, we propose that MF may affect the polyubiquitination process of SRPK1 (Figure 7C,D). Indeed, the polyubiquitination of SRPK1 was compromised in RCCEC treated with MF, whereas SPHINX31 did not exhibit this effect (Figure 7E; Figure S9D). Moreover, mRNA levels of *Srpk1* did not show a significant difference in RCCEC treated with either MF or SPHINX31 (Figure S9E). These results demonstrate that MF interacts with SRPK1 and is associated with reduced polyubiquitination, thereby stabilizing the protein.

We further evaluated the role of MF in PA‐induced SRPK1 protein degradation and HFD‐induced ED. In PA‐treated RCCEC, immunoblotting showed that MF restored SRPK1 protein levels in a dose‐dependent manner, paralleled by increased p53 and GPX4 expressions (Figure 7F). Additionally, MF treatment also reduced intracellular Fe^2+^ levels and rescued GSH depletion, indicating its effectiveness in suppressing ferroptosis induced by lipid accumulation (Figure 7G; Figure S9F,G). We explored the therapeutic potential of MF during the development of endothelial cell ferroptosis in the HFD‐fed rodent model (Figure S9H). Erectile function in the HFD‐fed, MF‐treated (HFD‐MF) group was significantly improved compared with the HFD‐fed, vehicle‐treated (HFD‐vehicle) group (Figure 7H). Moreover, serum lipid levels and fat compositions were marginally alleviated in the HFD‐MF group compared to the HFD‐vehicle group (Figure 7I; Figure S9I). In line with these observations, administration of MF effectively enhanced endothelial p53 and GPX4 expressions and reduced lipid peroxidation in rodent models (Figure 7J,K; Figure S9J). Our data also showed that injection of ferroptosis inhibitor Liproxstatin‐1 (Lip‐1) significantly attenuated HFD‐induced ED and decreased levels of 4‐HNE in the penile tissue (Figure S9K,L). Together, these findings suggest that MF mitigates endothelial cell ferroptosis by maintaining SRPK1 stability and regulating GPX4 expression.

## 3 Discussion

Multiple metabolic syndromes, including obesity, hypertension, and diabetes mellitus, disrupt endothelial homeostasis and initiate a cascade of pathological events that culminate in endothelial dysfunction, cell death, and an increased risk of cardiovascular diseases and ED [31, 32, 33]. Dyslipidemias, which are highly prevalent in patients with metabolic disorders, exacerbate endothelial injury, oxidative stress, and vascular remodeling, thereby compromising erectile function [34, 35, 36]. ED is recognized as a multifactorial condition arising from diverse pathophysiological insults, including metabolic disorders (e.g., diabetes mellitus, dyslipidemias), vascular endothelial dysfunction, aging, and nerve injury. Endothelial dysfunction is a common denominator across these etiologies, and several canonical mechanisms, including oxidative stress, nitric oxide (NO) deficiency, and inflammation, are central to its pathophysiology [2, 37]. Accumulating evidence suggests that multiple forms of programmed cell death play crucial roles in regulating erectile function, including autophagy, pyroptosis, and apoptosis [37, 38, 39, 40, 41]. Ferroptosis, a type of iron‐dependent programmed cell death characterized by phospholipid peroxidation, is increasingly recognized as a key mechanism in vascular diseases, and its inhibition may offer novel therapeutic strategies [42, 43]. The primary defense against ferroptosis is mediated by the GSH‐GPX4 axis, which detoxifies phospholipid hydroperoxides into phospholipid alcohols. Alternative protective pathways include the FSP1‐coenzyme Q10 axis and MBOAT1/2‐mediated cellular phospholipid remodeling [44]. In this study, we identify S‐palmitoylation as a critical regulator of SRPK1 protein stability and GPX4‐associated ferroptosis in the context of HFD exposure, particularly PA, a predominant saturated fatty acid in Western‐style diets. These findings reinforce the concept that an HFD, rich in various fatty acids, can contribute to the progression of cardiovascular events and related diseases [45, 46]. The ability of SRPK1 to regulate GPX4 and endothelial cell ferroptosis underscores its potential role in protecting against dyslipidemia‐related vascular diseases, which may serve as a therapeutic target beyond ED.

It is particularly intriguing that SRPK1, widely known for its serine/threonine kinase activity, is functionally associated with p53 phosphorylation at Ser15 in our study. Several protein kinases are involved in phosphorylating p53, such as ATM/ATR, CHK1, and CHK2, representing a well‐acknowledged mechanism of how p53 responds to DNA damage or external stress signals [47, 48, 49]. Moreover, p53 can control both canonical and non‐canonical ferroptotic processes as well as other cell death pathways, making it critical to elucidate which pathway predominates under specific conditions [25, 26]. The expression and activity of p53 are dynamically controlled by multilayered regulation at the DNA, RNA, and protein levels, in which numerous PTMs of p53 are influential in determining p53 activity [50]. Although ubiquitins typically target sites on the C‐terminal of p53 for protein degradation, our study identified a novel and conserved ubiquitination site at K139 within the DNA‐binding domain, which may also contribute to p53 stability regulation. Notably, this residue has been reported to undergo additional PTMs, including lactylation and ISGylation, suggesting potential regulatory crosstalk at this site [51, 52]. Distinct PTMs cooperate to regulate p53 stability, activity, and subcellular localization, which warrants further investigation into their crosstalk and collective impact on p53 function. In this study, we found that SRPK1‐associated p53 activation was accompanied by increased GPX4 expression in PA‐induced endothelial cell ferroptosis, leading to mitigated lipid peroxidation, reduced oxidative stress, and stable iron homeostasis. Therefore, a comprehensive understanding of complex mechanisms regulating p53 activity and function in endothelial biology could shed light on new strategic paths to therapeutic breakthroughs of vascular diseases.

Protein S‐palmitoylation has emerged as a versatile lipid modification that regulates diverse biological processes, including antiviral innate immunity, tumor metabolism, and cardiovascular dysfunction [7, 8, 9]. The intricate relationship between S‐palmitoylation and ubiquitination has been widely reported. For example, palmitoylation of membrane‐associated proteins often enhances their stability by promoting proper membrane localization and reducing proteasomal turnover [10, 53]. Notably, RIPK1 S‐palmitoylation, which depends on K63‐linked ubiquitination, has been revealed as a licensing modification for kinase activity, thereby inducing downstream cell death signaling in inflammatory diseases [54]. In endothelial cells, reversible S‐palmitoylation governs plasma membrane partitioning to maintain vascular maturity and coordinate vessel remodeling in peripheral artery diseases [55]. Moreover, PA accelerates endothelial injury through PKM2 S‐palmitoylation, impairing PKM2 tetramerization and inhibiting its pyruvate kinase activity, which in turn reduces endothelial glycolysis [8]. These observations underscore the importance of biochemical investigations into cellular lipid status, particularly the role of PA in modulating protein S‐palmitoylation. In this study, we demonstrated that exogenous PA, a primary substrate for acyl‐CoA synthetase, was associated with enhanced SRPK1 S‐palmitoylation and subsequent ubiquitination‐dependent protein degradation in endothelial cells. Furthermore, S‐palmitoylation mutation (C188S/C502S/C647S) or impaired enzymatic activity of ZDHHC24 and APT1 in this dynamically reversible PTM significantly impacts SRPK1 ubiquitination. Although AlphaFold‐based structural modeling did not predict major global conformational changes upon palmitoylation, our biochemical data indicate that S‐palmitoylation enhances the interaction between SRPK1 and MIB1, thereby facilitating SRPK1 degradation. Recently, Zheng et al. reported that palmitoylated NLRP3 enhanced NLRP3‐NEK7 interaction, promoting inflammasome assembly and activation [56]. Alternatively, SRPK1 palmitoylation may influence the accessibility or topology of ubiquitination site K494 or modulate ubiquitin linkage specificity, thereby promoting degradation. Previous research proposed that MAVS palmitoylation promotes its stabilization and activation by inhibiting K48‐linked but facilitating K63‐linked ubiquitination, providing novel insights into antitumor immunity [57]. Further structural and biochemical studies are required to delineate the precise molecular basis of SRPK1 S‐palmitoylation and its crosstalk with other PTMs.

Emerging evidence highlights the pivotal roles of palmitoyltransferases and acyl protein thioesterases in diverse pathological processes, including tumor cell ferroptosis, inflammasome activation, and antiviral immunity [7, 56, 58]. In endothelial cells, S‐palmitoylation serves as a key PTM that governs protein trafficking, signaling, and function, ultimately shaping vascular homeostasis and responses to injury or inflammation. Notably, the S‐palmitoylation of endothelial nitric oxide synthase (eNOS) facilitates its localization to caveolae, where its enzymatic activity is precisely regulated; subsequent depalmitoylation by APT1 modulates nitric oxide production and downstream signaling [59, 60]. Proteomic analyses have further unveiled that S‐palmitoylation affects the nuclear localization and enzymatic function of SOD1, while ZDHHC21‐mediated palmitoylation of PECAM1 is essential for its membrane targeting and cell‐cell adhesion [61]. Functionally, APT1 deficiency in endothelial cells disrupts vascular remodeling and leads to aberrant focal adhesion formation [55]. In addition, ZDHHC24 has been implicated in the S‐palmitoylation of MAVS and Akt, thereby modulating antiviral innate immunity and liver tumorigenesis, respectively [7, 62]. In this study, we identified ZDHHC24 and APT1 as key regulators of SRPK1 S‐palmitoylation and stability, underscoring their potential roles in endothelial function and cell viability. These findings provide mechanistic insights into how palmitoylation dynamics orchestrate endothelial homeostasis and suggest that modulating SRPK1 palmitoylation may represent a novel therapeutic strategy in vascular pathology.

Despite establishing a functional connection between reversible S‐palmitoylation and ubiquitination in regulating SRPK1 stability, several mechanistic questions remain unresolved. In particular, the structural basis underlying SRPK1 S‐palmitoylation and ubiquitination, as well as their potential crosstalk, has yet to be elucidated. Advanced structural approaches, such as cryo‐electron microscopy or integrative structural modeling, will be essential to delineate the conformational consequences of these modifications. Future investigations, including in vitro enzyme activity assays and click chemistry, are needed to confirm the roles of APT1 and ZDHHC24 in SRPK1 S‐palmitoylation as well as SRPK1 in p53 phosphorylation. We have not yet explored the precise ubiquitin linkage types at K494 in detail, possibly involving K48‐linked chains, given its established role in targeting proteins for degradation. Although molecular docking could predict key residues in SRPK1‐MF binding, further analysis, such as molecular dynamics simulations and surface plasmon resonance, is necessary to comprehensively characterize the binding kinetics and pharmacological properties. Notably, MF may act on additional targets and contribute to systemic lipid regulation, beyond its role in modulating endothelial cell ferroptosis. Additional research into other biochemical pathways implicated in ferroptosis is warranted in these settings, including the CoQ‐FSP1 axis, ACSL3/4, and MBOAT1/2. Addressing these issues could further determine whether modulation of SRPK1 offers therapeutic potential for addressing vascular complications associated with lipid accumulation and endothelial cell ferroptosis.

In conclusion, our study identifies a novel regulatory mechanism in which SRPK1 undergoes dynamic S‐palmitoylation and subsequent ubiquitination to control its protein stability, participating in p53 phosphorylation and GPX4‐dependent ferroptosis. These findings highlight PA as a key regulator of endothelial ferroptosis and identify SRPK1 as a potential therapeutic target for dyslipidemia‐associated ED. More broadly, the SRPK1‐p53‐GPX4 pathway may represent a convergent mechanism across multiple ED etiologies, including diabetes, aging, vascular disease, and nerve injury. Future investigations employing single‐cell and spatial transcriptomic approaches will be critical for delineating its mechanistic breadth and translational potential in vascular health and disease.

## 4 Materials and Methods

### 4.1 Animals and Ethics Statements

All animal experimental protocols used in this study were reviewed and approved by the Institutional Animal Care & Use Committee of Peking University First Hospital (Approval No. J2024054). Sprague‐Dawley (SD) rats and C57BL/6J mice were obtained from the Laboratory Animal Center of Peking University First Hospital. Animals were housed in a specific pathogen‐free environment, provided with sterile pellet food and water ad libitum, and maintained under controlled conditions of light (12/12 h light/dark cycle), temperature (22‐26°C), and humidity (50±10%).

### 4.2 Cell Lines and Cell Culture

Human umbilical vein endothelial cells (HUVEC, Cat# 8000, ScienCell) and rat corpus cavernosum endothelial cells (RCCEC, Cat# CP‐R133, Procell) were cultured in Endothelial Cell Medium (Cat# 1001, ScienCell) with 5% Fetal Bovine Serum (FBS), 1% Endothelial Cell Growth Supplement (Cat# 1052, ScienCell), and 1% Penicillin/Streptomycin. HEK293T (Cat# LV010, Applied Biological Materials; RRID: CVCL_0063) and derived cell lines were cultured in Dulbecco's Modified Eagle Medium (DMEM) with 10% FBS and 1% Penicillin/Streptomycin. All cells were cultured in a 37°C humidified incubator under 5% CO_2_ and tested negative for microbial contamination.

### 4.3 Animal Models, AAV9 Injection, and Pharmacological Treatment

For the erectile dysfunction rodent model induced by HFD, male mice and rats aged 6–8 weeks of the indicated genotypes were fed an HFD (60 kcal% fat, 20 kcal% protein, 20 kcal% carbohydrate) for 16 weeks. Animals maintained on a normal chow diet (NC, 10% kcal fat) served as controls.

For endothelial‐specific gene transduction, an AAV9 vector carrying SRPK1 under the control of an ICAM2 promoter (AAV‐Vector/AAV‐SRPK1, 2 × 10^10^ viral genomes per rat; WZ Bioscience Inc., Jinan, China) was administered by local injection into the corpus cavernosum. Following a 4‐week period for endothelial transduction, animals were subjected to an additional 16 weeks of HFD feeding. After a total experimental period of 20 weeks, the animals were sacrificed for further analyses.

For pharmacological intervention, male SD rats were fed an HFD for approximately 12 weeks and then randomized to receive either 4'‐O‐Methylochnaflavone (MF; 2.5 mg/kg/day) or vehicle by oral administration, while HFD feeding continued for an additional 4 weeks. In parallel, male mice received daily intraperitoneal injections of Liproxstatin‐1 (Lip‐1; 10 mg/kg/day) or vehicle. At the end of treatment, animals were euthanized, and tissues were collected for subsequent analyses.

### 4.4 Generation of Genetically Modified Mice

Genetically modified mice with *Srpk1* endothelial‐specific knock‐in (*Srpk1*‐EKI) were successfully generated by CRISPR/Cas9‐mediated gene editing and Cre‐loxP recombination technology. Briefly, a loxP‐flanked transcriptional STOP cassette followed by the full‐length *Srpk1* coding sequence (CDS) was inserted into the *Gt(ROSA)26Sor* (*Rosa26*) locus on a C57BL/6J background to generate *Rosa26^LSL‐Srpk1^* mice. Endothelial‐specific activation of *Srpk1* expression was achieved by crossing *Rosa26^LSL‐Srpk1^* mice with *Cdh5*‐Cre transgenic mice, in which Cre recombinase is selectively expressed in endothelial cells. Unless otherwise indicated, *Srpk1*‐EKI mice refer to *Rosa26^LSL‐Srpk1^*; *Cdh5*‐Cre+ animals. Littermate *Rosa26^LSL‐Srpk1^*; *Cdh5*‐Cre‐ mice were used as control (*Srpk1*‐Ctrl) throughout the study.

### 4.5 Measurement of Erectile Function

Animals from each group were anesthetized via intraperitoneal injection of 2.5% Avertin (2,2,2‐tribromoethanol; Sigma–Aldrich). The cavernous nerve was carefully dissected and exposed, and then a bipolar platinum wire electrode was positioned around it for electrical stimulation. Erectile function was evaluated using the Biopac Student Lab System (BioPac Systems Inc., Goleta, CA, USA) with standardized parameters: 5 V voltage, 20 Hz frequency, 1 ms pulse width, and 1‐min stimulation duration. During tumescence, maximal intracavernous pressure (ICP) was continuously recorded. Erectile response was normalized by calculating the ratio of maximum ICP to mean systolic blood pressure (MSBP) or mean arterial pressure (MAP), ensuring consistent comparison across groups.

### 4.6 Serological Assay

Triglyceride (TG), total cholesterol (TC), low‐density lipoprotein cholesterol (LDL‐C), and high‐density lipoprotein cholesterol (HDL‐C) levels were measured in animals to evaluate serum lipid profile on a Chemray 800 Automatic Analyzer (Rayto, Shenzhen, China) according to the manufacturer's instructions.

### 4.7 Immunoblot Analysis and Nuclear‐Cytoplasmic Fractionation

Cells were harvested and lysed using ice‐cold RIPA buffer containing a protease inhibitor cocktail and phosphatase inhibitors for 30 min on ice. The resulting cellular lysates were subjected to sodium dodecyl sulfate‐polyacrylamide gel electrophoresis (SDS‐PAGE) and then transferred onto polyvinylidene difluoride (PVDF) membranes. The membranes were blocked and incubated with the appropriate primary antibodies, followed by incubation with HRP‐conjugated secondary antibodies. The information about antibodies was shown in Table S4. Immunoreactive bands were detected using an enhanced chemiluminescence system (Syngene G‐Box, Syngene, Cambridge, UK), and band intensities were quantified using ImageJ software to assess relative protein expression levels. Nuclear and cytoplasmic protein compartments were extracted with the NE‐PER Nuclear and Cytoplasmic Extraction Reagent kit (ThermoFisher Scientific, 78833) following standard manufacturer's instructions. After extraction, nuclear and cytoplasmic extracts were separated by SDS‐PAGE and analyzed by immunoblotting as above.

### 4.8 Immunohistochemistry

Paraffin‐embedded sections of rodent corpus cavernosum tissues were processed for immunostaining. Endogenous peroxidase activity was quenched by incubating the sections in 3% H_2_O_2_ for 30 min at 37°C. Antigen retrieval was then performed using microwave treatment. The sections were subsequently blocked with 5% bovine serum albumin (BSA) for 60 min at room temperature. Primary antibodies were applied to detect the target proteins, followed by incubation with horseradish peroxidase (HRP)‐conjugated secondary antibodies. Signal development was achieved using diaminobenzidine (DAB), and the slides were counterstained with hematoxylin, dehydrated, and mounted for examination via bright‐field microscopy.

### 4.9 Immunofluorescence

Fixed tissues were incubated in 30% sucrose in PBS overnight at 4°C, followed by embedding in OCT mounting compound (TissueTek, Sakura). Frozen tissues were cut into 5‐µm‐thick sections using a Leica CM1860 cryostat. Tissue sections were postfixed with 1% paraformaldehyde in PBS for 10 min. Sections were incubated with blocking solution (5% goat serum and 0.3% Triton X‐100 in PBS) for 30 min and then incubated with primary antibodies overnight at 4°C. The next day, sections were incubated with appropriate fluorophore‐conjugated secondary antibodies for 2 h at room temperature. Nuclei were labeled by staining with DAPI for 5 min. Images were obtained using a DFC425 color microscope (Leica) and processed with ImageJ.

### 4.10 Immunoprecipitation

Cells were lysed in ice‐cold IP Lysis Buffer supplemented with a protease inhibitor cocktail. The lysis was carried out for 30 min at 4°C, followed by centrifugation at 12 000 *xg* for 10 min at 4°C. Protein concentrations were quantified using the BCA assay (Beyotime). For Myc‐tagged proteins, lysates were incubated with anti‐c‐Myc magnetic beads (Cat# HY‐K0206, MedChemExpress, China) at 4°C overnight. For endogenous protein immunoprecipitation, lysates were incubated with primary antibodies and protein G magnetic beads at 4°C overnight. Immunocomplexes were washed three times with ice‐cold washing buffer and then eluted in sample buffer containing 1% SDS at 95°C for 10 min. The eluted proteins were subsequently analyzed by SDS‐PAGE and immunoblotting.

### 4.11 Real‐Time Quantitative Reverse‐Transcription PCR

Total RNA was extracted using 1 mL of TRIzol reagent following the manufacturer's instructions. A total of 1 µg of RNA was reverse‐transcribed into cDNA using the FastKing cDNA synthesis kit (TIANGEN). Real‐time quantitative PCR was performed on a Real‐Time PCR System using BlasTaq 2X qPCR MasterMix (Cat# G891, Applied Biological Materials). The relative gene expression levels were calculated using the 2^^−ΔΔCt^ method. qRT‐PCR data were collected from at least three independent experiments, with each experiment performed in triplicate. The oligonucleotide primer sequences are provided in Table S5.

### 4.12 Cycloheximide (CHX) Chase Assay

Cells were treated with cycloheximide (Cat# HY‐12320, MedChemExpress) and collected at the indicated time points. Changes in protein levels were analyzed by immunoblot. Quantification of protein abundance was performed using ImageJ and plotted accordingly.

### 4.13 Small Interfering RNA (siRNA) and Plasmid Transfection

Cells were transfected with small interfering RNA (siRNA) targeting ZDHHC‐protein acyltransferases (General Biology, Anhui, China) using JetPRIME Transfection Reagent (Cat# 101000046, Polyplus, France) following the manufacturer's instructions. For plasmid transfection, HEK293T cells were transfected with the desired plasmid using PEI Transfection Reagent (Cat# 23966, Polysciences) in Opti‐MEM (Cat# 31985070, Invitrogen) according to the manufacturer's protocol. After 12–18 h of incubation, the medium was replaced with complete medium, and cells were incubated for an additional 48 h before further experiments.

### 4.14 Lentivirus Production and Infection

For lentiviral production, HEK293T cells were transfected with either pLKO.1‐puromycin vector carrying specific shRNA or desired pLenti‐puromycin vector with pMD2.G and psPAX2 plasmids for 48 h. Harvested supernatant media from transfected HEK293T cells was filtered through a 0.45 µm filter. Filtered media containing lentiviral particles were used to infect cells in the presence of polybrene (8 µg/mL). After 24 h of incubation with the virus, the medium was replaced with fresh medium and selected with 1 µg/mL puromycin.

### 4.15 Cell Viability Assay

Cells were seeded at an appropriate cell density and subjected to the indicated treatments as described in individual experiments. At the end of treatment, cell viability was measured by CCK8 Cell Viability Assay (Cat# KGA317, Keygen Biotech) with a microplate reader (ThermoFisher Scientific). Relative viability was calculated by normalizing the absorbance at 450 nm of treatments to no treatment control.

### 4.16 Acyl‐Biotin Exchange (ABE) Assay

The ABE assay was performed as described previously with minor modifications. Briefly, HEK293T cells transfected with the indicated plasmids or siRNAs were harvested 72 h post‐transfection and washed with cold PBS. Cell lysates were collected and incubated with N‐ethylmaleimide (NEM) at 4°C overnight in the presence of a protease inhibitor cocktail. Proteins were precipitated using a methanol‐chloroform mixture and subsequently dissolved in lysis buffer (pH 7.4, 50 mm Tris‐HCl, 1 mm EDTA, 150 mm NaCl, and 1% NP‐40) containing 0.8 M hydroxylamine (HAM; Cat# 438227, Sigma–Aldrich) at room temperature for 1 h. Parallel control reactions were performed in the absence of hydroxylamine to confirm specificity. The reprecipitated proteins were then incubated with Biotin‐HDPD (20 mm; Cat# GC11037, GLPBio) and enriched using pre‐washed streptavidin magnetic beads with rotation at 4°C overnight. The beads were washed, and the proteins were eluted using an elution buffer at 95°C for 10 min. Finally, the eluted proteins were separated by SDS‐PAGE and analyzed by immunoblotting.

### 4.17 Cellular Fe^2+^ Measurement

For cellular Fe^2+^ measurement, endothelial cells were seeded into 96‐well cell culture plates. After 24 h, the cells were washed twice with HBSS. Serum‐free DMEM containing 1 µm FerroOrange probe (Cat# F374, Dojindo) was then added to each well, and the plates were incubated at 37°C in the dark for 30 min. Following incubation, the cells were washed and immediately analyzed using a multi‐plate reader.

### 4.18 Glutathione Measurement

For glutathione measurement, endothelial cells were seeded into a six‐well culture plate. After 18 h, cells were collected by scraping and processed for glutathione (GSH) measurement using the Glutathione Assay Kit (Cat# S0052, Beyotime) according to the manufacturer's protocol. GSH concentrations were calculated based on a standard curve and normalized to total protein content. Three independent biological replicates were performed.

### 4.19 Intracellular ROS Measurements

Cellular reactive oxygen species (ROS) levels were assessed using dichloro‐dihydro‐fluorescein diacetate (DCFH‐DA) (Cat# S0033, Beyotime) staining. Cells were incubated with 10 µm DCFH‐DA for 30 min, and then images were acquired using a fluorescence microscope. The intensity was quantified using ImageJ software.

### 4.20 Lipid Peroxidation Measurement

Endothelial cells were seeded into six‐well plates at 50% confluence before different treatments. Intracellular lipid peroxidation was measured using the Lipid Peroxidation MDA Assay Kit (Cat# S0131S, Beyotime) according to the manufacturer's instructions. Thiobarbituric acid (TBA) was added to the supernatants of cell homogenates to form a TBA‐malondialdehyde (MDA) adduct, which was then analyzed spectrophotometrically at 532 nm to quantify MDA levels.

### 4.21 Molecular Docking

Molecular docking was performed using AutoDock Vina. The docking procedure required input files for the receptor macromolecule (PDB ID: 5MY8) and ligands from a flavonoid library (Cat# HY‐L068, MedChemExpress), along with a configuration file specifying the following parameters: gridbox center coordinates as x = 16.571, y = 46.733, and z = 31.615; gridbox dimensions of 40 Å for the X, Y, and Z axes; an energy range of 4 kcal/mol; exhaustiveness of 12; and num_modes of 10. Perl scripts were employed to generate these configuration files and to execute the AutoDock Vina program.

### 4.22 Biotin Pull‐Down Assay

The synthesis of biotin‐MF was performed by WayenBio, and the compound was supplied with an approved Certificate of Analysis. Biotin‐MF pull‐down assays were conducted using the Biotinylated Protein Interaction Pull‐Down Kit (Cat# 21115, ThermoFisher Scientific). Briefly, immobilized streptavidin was pre‐incubated with either free biotin or biotin‐labeled MF for 30 min at 4°C. After adequate blocking and washing, the “bait protein” complex was incubated with endothelial cell lysates or recombinant SRPK1 protein (Cat# 12249‐H20B, SinoBiological) under rotation overnight at 4°C. Following four washes with the provided Wash Buffer, the beads were eluted and then analyzed by immunoblotting.

### 4.23 Ubiquitinated Proteome Mass Spectrometry

Endothelial cells were cultured to 80–90% confluence and treated with fatty acid‐free BSA or PA as indicated. Cells were washed with ice‐cold PBS and lysed in buffer (50 mm Tris‐HCl, 150 mm NaCl, 1% NP‐40, 1 mm EDTA) supplemented with protease inhibitors, phosphatase inhibitors, and deubiquitinase inhibitors. Lysates were clarified by centrifugation at 4°C, and protein concentrations were determined using a BCA assay. Equal amounts of protein were subjected to enrichment of ubiquitinated proteins by incubation with anti‐ubiquitin antibody‐conjugated Protein A/G magnetic beads overnight at 4°C with gentle rotation. After extensive washing, bound proteins were eluted for downstream analysis.

Eluted proteins were reduced with dithiothreitol, alkylated with iodoacetamide, and digested overnight with sequencing‐grade trypsin (enzyme‐to‐substrate ratio 1:50). Resulting peptides were acidified with 0.1% formic acid, desalted using C18 solid‐phase extraction, and separated by nano‐liquid chromatography on an EASY‐nLC system coupled to a Q Exactive mass spectrometer operated in data‐dependent acquisition mode. Raw data were processed using Proteome Discoverer and searched against the UniProt protein database. Carbamidomethylation of cysteine was set as a fixed modification, whereas oxidation of methionine and ubiquitin‐derived lysine modifications were specified as variable modifications. Peptide‐spectrum matches were filtered to achieve a false discovery rate of 1% at both peptide and protein levels.

### 4.24 RNA Sequencing

Total RNA was isolated from samples using TRIzol reagent (Invitrogen) per the manufacturer's protocol. Subsequently, mRNA‐seq libraries were constructed with the mRNA‐seq Lib Prep Kit (Cat# RK20302, ABclonal) and sequenced on an Illumina NovaSeq 6000 platform. The raw sequencing files were initially assessed for quality using FastQC (version 0.11.7) and subsequently trimmed for adapter sequences with Cutadapt (version 2.7). The cleaned reads were then aligned to the human (GRCh38.102/hg38) and rat (Rnor_6.0.102/rn6) reference genomes using Hisat2 (version 2.0.5). Transcript assembly was performed using Stringtie, with gene abundance quantified as FPKM. Differential expression analysis was conducted and further interpreted using Gene Ontology (GO) and Kyoto Encyclopedia of Genes and Genomes (KEGG) pathway enrichment. All gene set files were sourced from the GSEA website (www.broadinstitute.org/gsea), and Gene Set Enrichment Analysis (GSEA) was carried out using the GSEA software (version 4.3.2).

### 4.25 Statistical Analysis

All statistical analyses were performed using GraphPad Prism 9 (GraphPad Software, La Jolla, CA). Data are presented as mean ± SEM from at least three independent experiments. Prior to any parametric testing, the normal distribution of the data was confirmed using the Shapiro‐Wilk test and the Kolmogorov‐Smirnov test. Statistical significance was evaluated using either a two‐tailed Student's t‐test or one‐way/two‐way analysis of variance (ANOVA) with Tukey's multiple comparisons, unless otherwise specified. A *p*‐value of less than 0.05 was considered statistically significant.

## Author Contributions

R.‐L.G., X.‐S.L., and Y.‐M.Y. contributed to the conceptualization of the study; R.‐L.G., X.‐S.L., and H.J. were responsible for project administration; R.‐L.G. and X.‐S.L. provided supervision; R.‐L.G. acquired funding; X.‐H.T., K.‐F.L., M.‐C.X., F.‐Z.Z., H.‐G.Y., Z.Z., P.‐C.G., and G.‐Q.X. conducted the investigation; X.‐H.T. and K.‐F.L. performed the formal analysis; X.‐H.T. and K.‐F.L. prepared the original draft of the manuscript; and X.‐H.T., K.‐F.L., and R.‐L.G. reviewed and edited the manuscript. All authors reviewed and approved the final version of the manuscript.

## Conflicts of Interest

The authors declare no conflicts of interest.

## Supporting information




**Supporting File 1**: advs74074‐sup‐0001‐SuppMat.pdf.

**Supporting File 2**: advs74074‐sup‐0002‐TablesS1‐S5.xlsx.

## Data Availability

All supporting data are available from corresponding authors upon reasonable request.
