# Supplementary material for: Palmitoylation‐Mediated Ubiquitination of SRPK1 Regulates Ferroptosis in High‐Fat‐Induced Erectile Dysfunction
Source: Adv Sci (Weinh). 2026 Jan 29;13(19):e13796. doi: 10.1002/advs.202513796 (PMC13045379; doi:10.1002/advs.202513796)
Supplement: Supplementary file 1 — Supporting File 1: advs74074‐sup‐0001‐SuppMat.pdf. [file ADVS-13-e13796-s002.pdf]

Supplemental Materials for

Palmitoylation-mediated Ubiquitination of SRPK1 Regulates Ferroptosis in High-Fat-Induced Erectile Dysfunction

Xiao-Hui Tan *et al.*

Corresponding authors: guanruili@bjmu.edu.cn (Rui-Li Guan); jianghui@bjmu.edu.cn (Hui Jiang); pineneedle@sina.com (Xue-Song Li)

The PDF file includes:

Figs. S1 to S9

Other Supplementary Material for this manuscript includes the following:

Tables S1 to S5

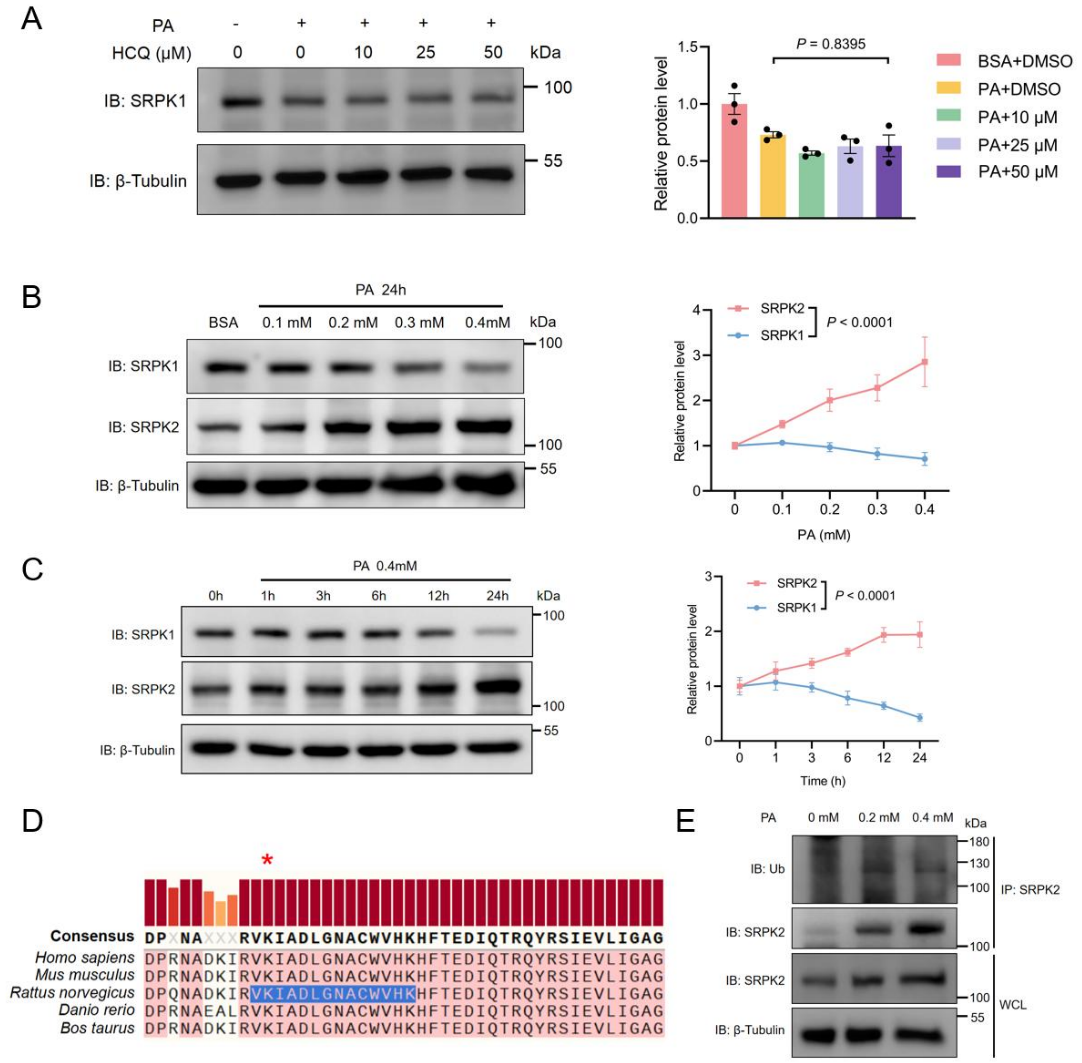

Figure S1. PA facilitates the ubiquitination-dependent degradation of SRPK1, related to Figure 1

A. RCCEC were treated with PA (0.25 mM) and hydroxychloroquine (HCQ), an inhibitor of lysosomal activity and autophagy, at the indicated concentrations (0-50 μM). Data represent the mean ± s.e.m. of three independent experiments. *P* values were calculated by one-way ANOVA followed by Tukey's multiple comparison test.

B. RCCEC were treated with PA at the indicated concentrations (0-0.4 mM, 24 h). Data represent the mean ± s.e.m. of three independent experiments. *P* values were calculated by two-way ANOVA analysis.

C. RCCEC were treated with PA at the indicated time (0-24 h, 0.4 mM). Data

represent the mean ± s.e.m. of three independent experiments. *P* values were calculated by two-way ANOVA analysis.

D. Sequence conservation of the ubiquitinated sequence (blue) and site (red asterisk) of SRPK2 across different species. The consensus with a threshold of more than 95% was highlighted (pink), with the sequence conservation shown in colored bars.

E. RCCEC were treated with MG-132 (10 μM, 6 h) and PA (0 mM, 0.2 mM, 0.4 mM) as indicated. Lysates were immunoprecipitated with anti-SRPK2, and immunoblot analysis was performed to analyze the levels of ubiquitination.

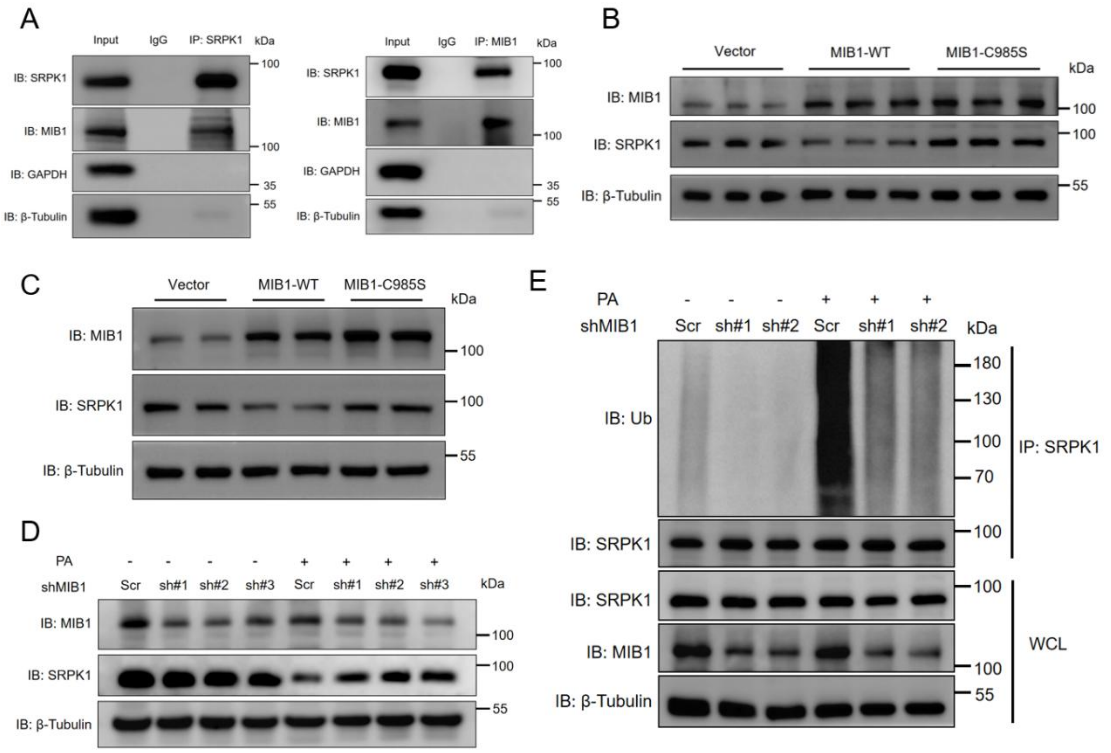

Figure S2. E3 ubiquitin ligase MIB1 regulates ubiquitination-dependent degradation of SRPK1, related to Figure 2

A. Lysates from RCCEC treated with PA and MG-132 were immunoprecipitated with anti-SRPK1 or anti-MIB1. Immunoblots were performed to analyze the interaction between MIB1 and SRPK1.

B. RCCEC were transfected with DNA constructs as indicated for 96h. The protein levels of SRPK1 and MIB1 were analyzed by western blot (n = 3 biological replicates).

C. The protein levels of SRPK1 and MIB1 were analyzed in HEK293T cells with overexpression of MIB1-WT or MIB1-C985S (n = 2 technical replicates).

D. RCCEC were transfected with scrambled shRNA (Scr) or MIB1 shRNAs (shMIB1 #1, shMIB1 #2, shMIB1 #3) for 48h, and then treated with BSA or PA (0.25 mM). The protein levels of SRPK1 and MIB1 were analyzed by western blot.

E. RCCEC were first transfected with scrambled shRNA (Scr) or MIB1 shRNAs (shMIB1 #1, shMIB1 #2) for 48h, then treated with BSA or PA (0.25 mM) and MG-132 (10 μM, 6h). Lysates were immunoprecipitated

with anti-SRPK1, and western blots were performed to analyze the levels of ubiquitination.

A

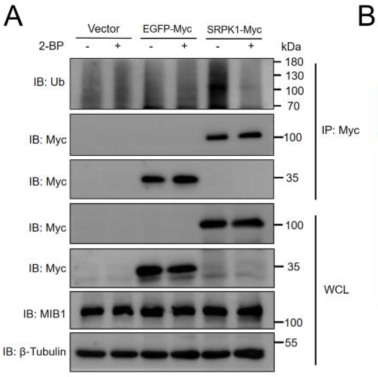

B

Bioinformatic database	Gene name	Species	Predicted candidate site	Amino acid sequence	Score
GPS-Palm	*SRPK1*	*Homo sapiens*	C188	KSNYQGLPLPCVKKIIQQV	0.8266
			C502	KVKIADLGNACWVHKHFTE	0.6214
			C647	IPEKRATAAECLRHPWLNS	0.7818
	Srpk1	*Mus musculus*	C188	KSNYQGLPLPCVKKIIQQV	0.8523
			C495	QVKIADLGNACWVHKHFTE	0.7013
			C640	MPEKRATAAECLRHPWLNS	0.7641

C

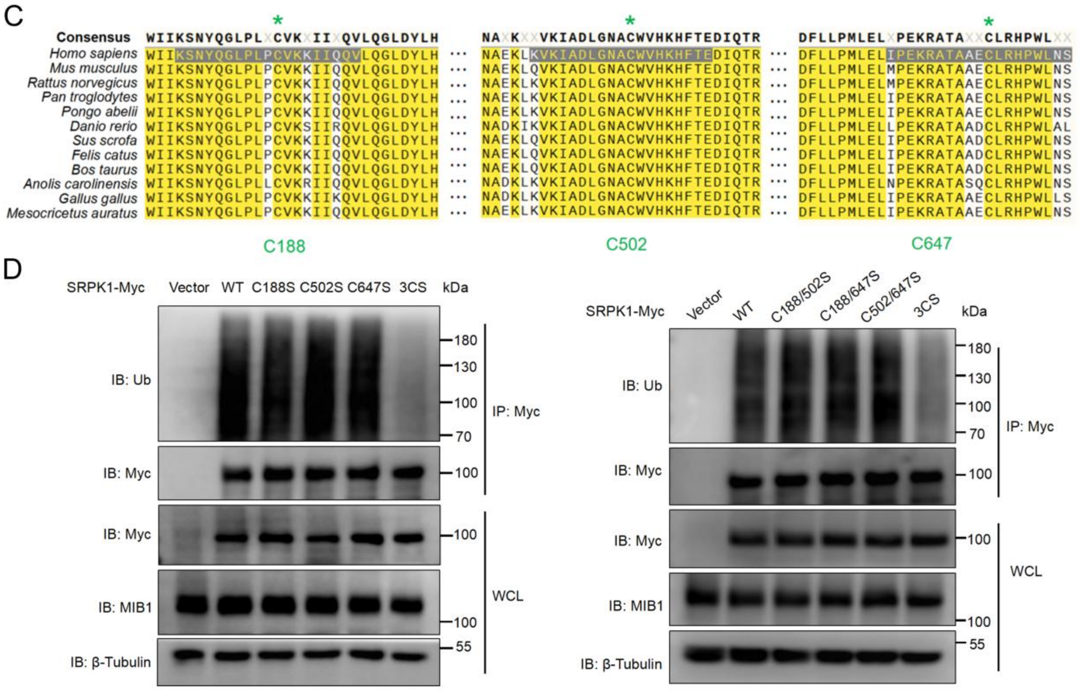

E F

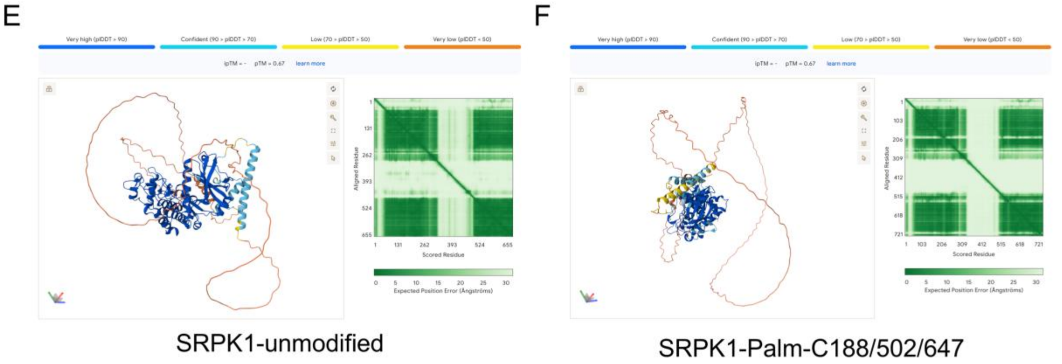

Figure S3. S-palmitoylation of SRPK1 at Cys188/502/647 is essential for its degradation, related to Figure 3

A. HEK293T cells transfected with indicated DNA constructs were treated with 2-BP (20 μM, 24h) and MG-132 (10 μM, 6h). Lysates were immunoprecipitated with anti-Myc to analyze the levels of ubiquitination via

immunoblot analysis.

B. Prediction of SRPK1 S-palmitoylation sites (green) by the GSP-Palm software.

C. Sequence conservation of the predicted palmitoylated sequence (gray) and site (green asterisk) of SRPK1 across different species. The consensus with a threshold of more than 95% was highlighted (yellow).

D. HEK293T cells were transfected with DNA constructs and treated with MG-132 (10 μM, 6h). Lysates were immunoprecipitated with anti-Myc to analyze the levels of ubiquitination via immunoblot analysis.

E. AlphaFold prediction was made for a full-length human SRPK1 amino acid sequence (Uniprot accession number: Q96SB4) without any modifications (SRPK1-unmodified). This prediction was colored by the predicted local distance difference test (pLDDT) as a measure of confidence or disorder.

F. AlphaFold prediction was made for a full-length human SRPK1 amino acid sequence (Uniprot accession number: Q96SB4) with three key residues palmitoylated simultaneously (SRPK1-Palm-C188/502/647), which was colored by the predicted local distance difference test (pLDDT) as a measure of confidence or disorder.

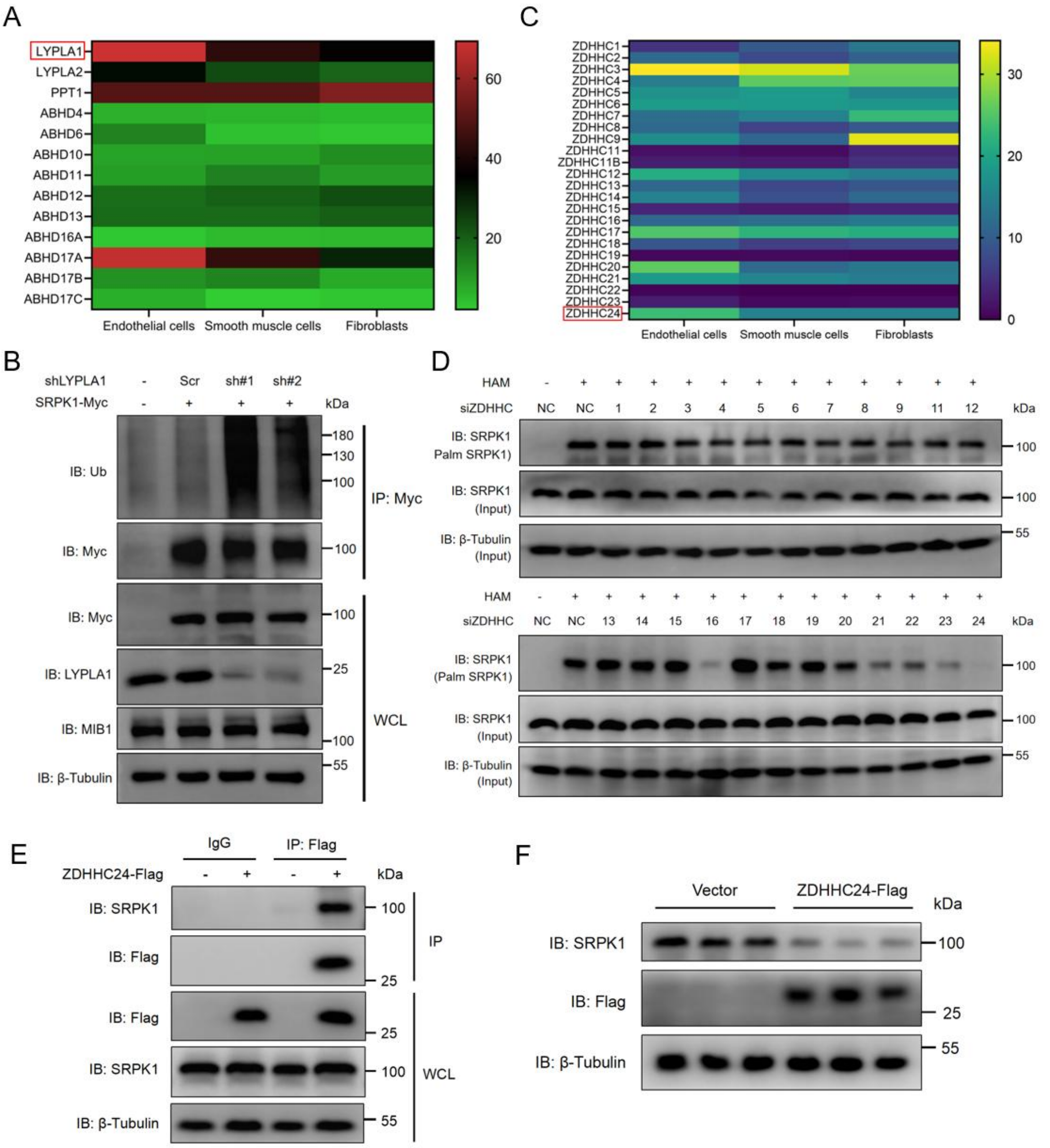

Figure S4. APT1 and ZDHHC24 mediate S-palmitoylation of SRPK1, related to Figure 4

- **A.** Heatmap of gene expression of deacylation enzymes in different cell types (Data from CellPalmSeq and Human Protein Atlas).
- **B.** HEK293T cells with knockdown of LYPLA1 were transfected with DNA constructs as indicated and treated with MG-132 (10 μM, 6h). Lysates were immunoprecipitated with anti-Myc, and immunoblot analysis was performed to analyze the levels of ubiquitination.
- **C.** Heatmap of ZDHHCs gene expression in different cell types (Data from

CellPalmSeq and Human Protein Atlas).

D. After transfection with the indicated siRNAs in HEK293T cells for 48h, the cellular lysates were subjected to the ABE assay.

E. Lysates from HEK293T cells transfected with DNA constructs as indicated and treated with MG-132 (10 μM, 6h) were immunoprecipitated with anti-Flag. Immunoblot analysis was performed to analyze the interaction between ZDHHC24-Flag and SRPK1.

F. HEK293T cells were transfected with indicated DNA constructs. The protein levels of SRPK1 and ZDHHC24-Flag were analyzed by immunoblot analysis (n = 3 biological replicates).

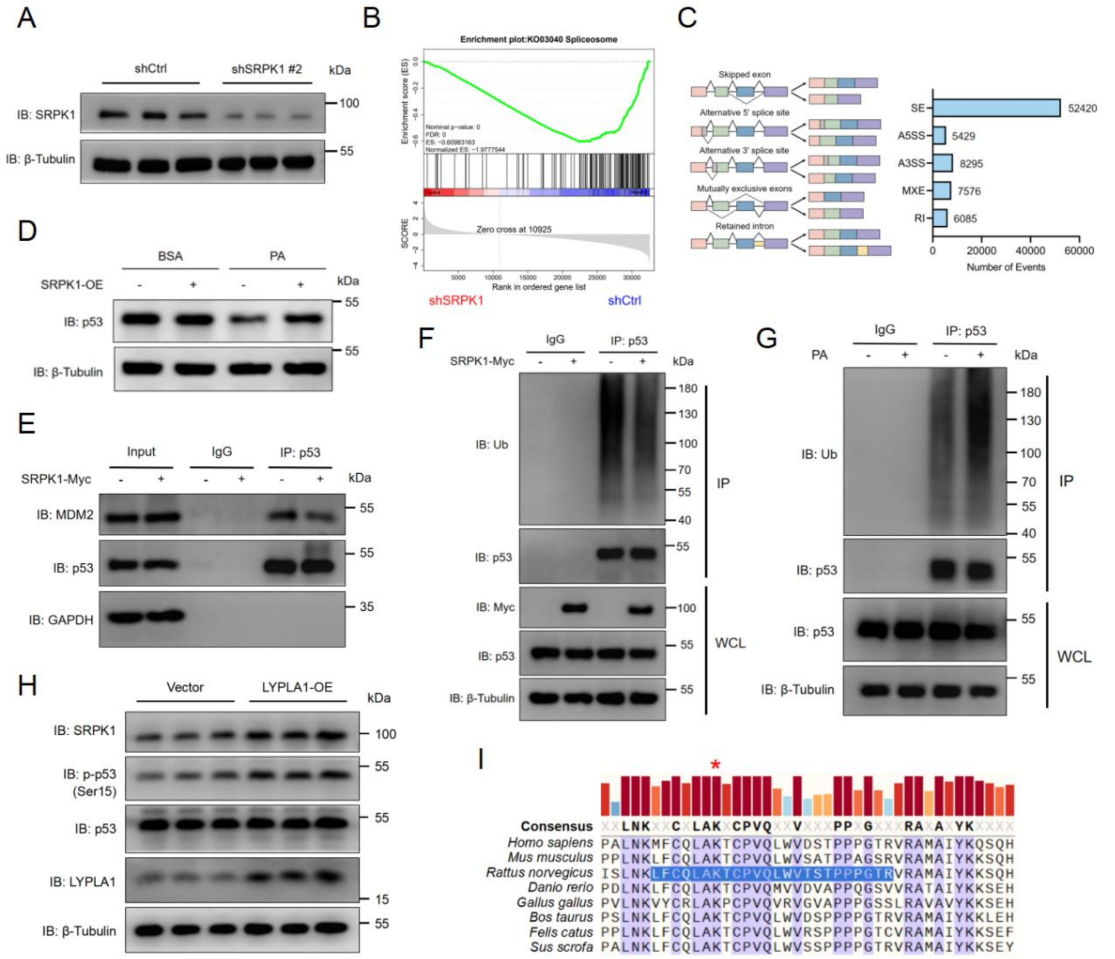

Figure S5. SRPK1 mediates p53 phosphorylation at Ser15, related to Figure 5

A. Immunoblot analysis confirming stable knockdown of SRPK1 in HEK293T cells (n = 3 biological replicates).

B. Gene Set Enrichment Analysis (GSEA) was performed on a gene set related to the spliceosome pathway using the RNA-seq data (GEO: GSE283448).

C. Schematic diagram of different types of alternative splicing events (left) and bar chart (right) of the number of different types of alternative splicing events using the RNA-seq data (GEO: GSE283448).

D. RCCEC overexpressing SRPK1 were treated with BSA or PA (0.25 mM), and whole-cell lysates were analyzed for immunoblot detection of p53.

E. HEK293T cells were transfected with indicated DNA constructs and treated with MG-132 (10 μM, 6h). Lysates were immunoprecipitated with

anti-p53. Immunoblot analysis was performed to analyze the interaction between p53 and MDM2.

F. Lysates from HEK293T cells transfected with indicated DNA constructs and treated with MG-132 (10 μM, 6h) were immunoprecipitated with anti-p53. Immunoblot analysis was performed to analyze the levels of ubiquitination.

G. RCCEC were treated with PA (0.25 mM) and treated with MG-132 (10 μM, 6h). Lysates were immunoprecipitated with anti-p53, and immunoblot analysis was performed to analyze the levels of ubiquitination.

H. Immunoblot analysis of p-p53 (Ser15), p53, and SRPK1 in HEK293T cells with overexpression of LYPLA1.

I. Sequence conservation of the ubiquitinated sequence (blue) and site (red asterisk) of p53 across different species. The consensus with a threshold of more than 95% was highlighted (purple), with the sequence conservation shown in colored bars.

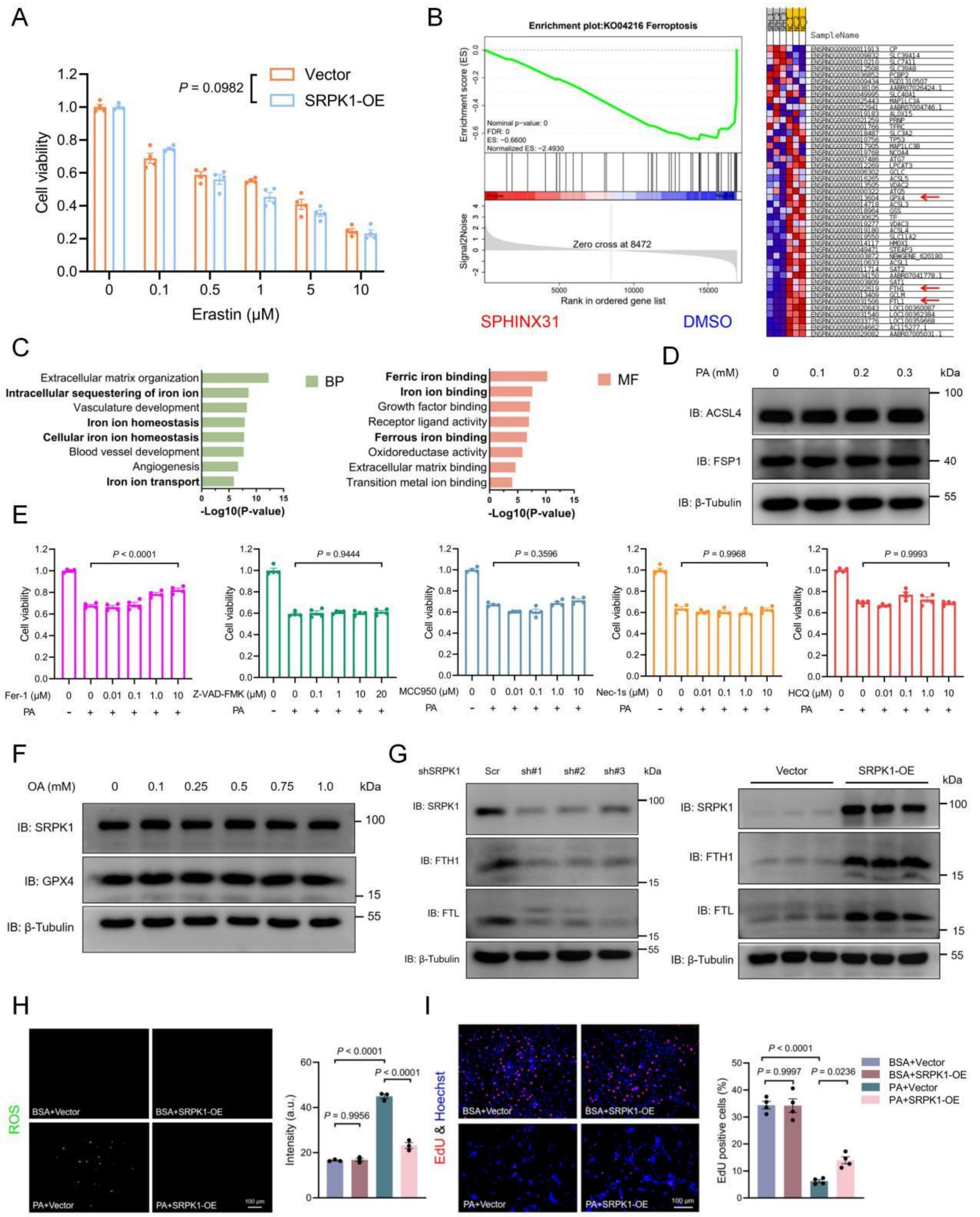

Figure S6. SRPK1 attenuates PA-associated endothelial cell ferroptosis, related to Figure 6

A. Cell viability was measured after treating RCCEC overexpressing SRPK1 with Erastin, an inhibitor of cystine-glutamate antiporter, for 24 h. Data represent the mean ± s.e.m. of 4 wells of a 96-well plate. *P* values were

calculated by two-way ANOVA analysis.

B. Gene Set Enrichment Analysis (GSEA) on a gene set related to the ferroptosis pathway in RCCEC treated with SPHINX31 or DMSO (GEO: GSE211557), showing the down-regulated expressions of GPX4, FTH1, and FTL1 (red arrows).

C. Gene Ontology (GO) enrichment analysis of differentially expressed genes identified in RNA-seq data (GEO: GSE211557). BP, biological processes; MF, molecular functions.

D. RCCEC were treated with PA at the indicated concentration. The expressions of FSP1 and ACSL4 were analyzed via immunoblot analysis.

E. Cell viability of RCCEC treated with PA (0.25 mM) along with different cell death inhibitors against ferroptosis (ferrostatin-1 [Fer-1]), apoptosis (Z-VAD-FMK), pyroptosis (MCC950), necroptosis (Nec-1s), and autophagy (HCQ) at the indicated concentrations. Data represent the mean ± s.e.m. of 4 wells of a 96-well plate. *P* values were calculated by one-way ANOVA analysis.

F. RCCEC were treated with oleic acid (OA) at the indicated concentration, and whole-cell lysates were analyzed for SRPK1 and GPX4.

G. Lysates from HEK293T cells were transfected with indicated shRNAs (left) and DNA constructs (right, n = 3 biological replicates). The expressions of FTH1/FTL were analyzed via immunoblot analysis.

H. Intracellular ROS production (left) and semi-quantification (right) in endothelial cells of the indicated group. Scale bars, 100 μm.

I. EdU (red) and Hoechst (blue) staining in endothelial cells of the indicated group. The percentage of EdU-positive cells was quantified by ImageJ. Scale bars, 100 μm.

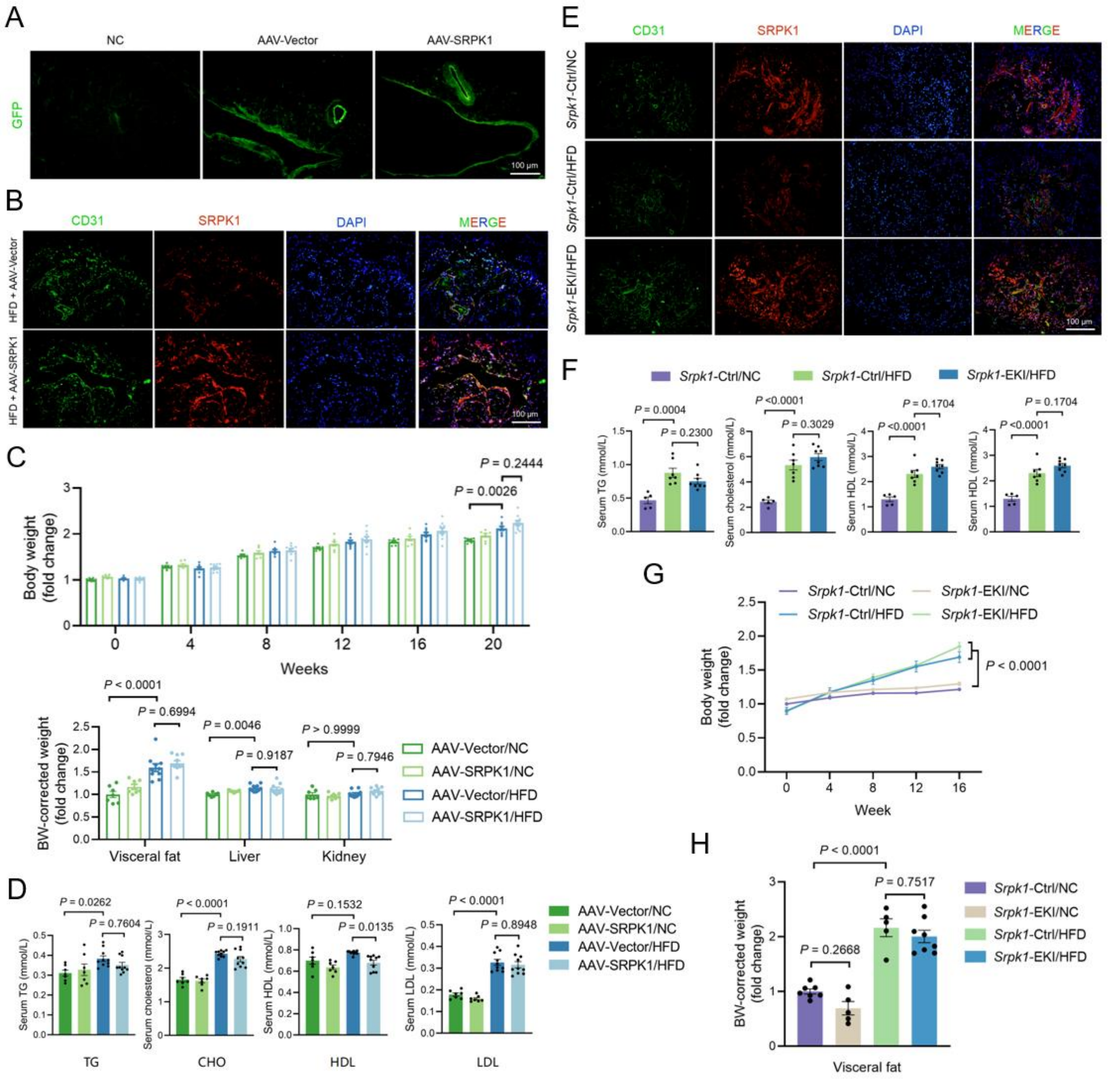

Figure S7. Endothelial-specific overexpression of SRPK1 alleviates HFD-induced erectile dysfunction, related to Figure 6

A. Representative IF images of GFP expression in the corpus cavernosum tissues of the negative control (NC) group, AAV-Vector group, and the AAV-SRPK1 group.

B. Images of immunofluorescence staining of corpus cavernosum tissues from HFD-fed rats for SRPK1 (red) and CD31 (green). Scale bars, 100 μm. Nuclei, DAPI, blue.

C. Body weight (BW) and BW-corrected weight of organs in male rats from the indicated group (n = 6-10 rats/group).

D. Serum lipid profile of male rats from the indicated group, as determined by assay (n = 6-10 rats/group).

- **E.** Images of immunofluorescence staining of corpus cavernosum tissues from mice for SRPK1 (red) and CD31 (green). Scale bars, 100 μm. Nuclei, DAPI, blue.
- **F.** Serum lipid profile of male mice from the indicated group, as determined by assay (n = 5-8 mice/group).
- **G.** Body weight (BW) in male mice from the indicated group (n = 5-8 mice/group).
- **H.** BW-corrected weight of visceral fat in male mice from the indicated group (n = 5-8 mice/group).

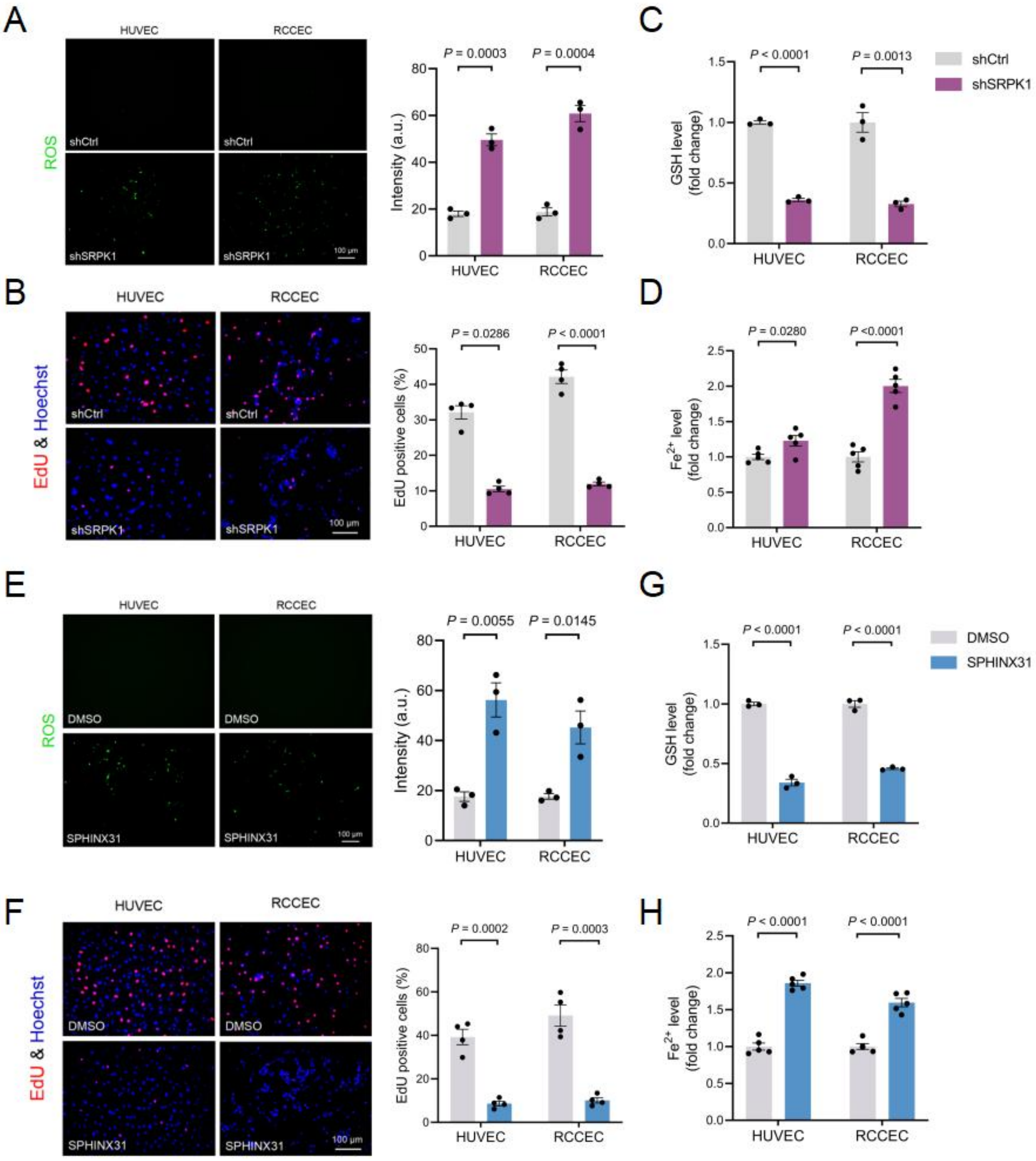
A
HUVEC
RCCEC
ROS
shCtrl
shSRPK1
100 μm
P = 0.0003
P = 0.0004
Intensity (a.u.)
B
EdU & Hoechst
EdU positive cells (%)
P = 0.0286
P < 0.0001
C
GSH level
(fold change)
P < 0.0001
P = 0.0013
D
Fe^{2+} level
(fold change)
P = 0.0280
P <0.0001
E
DMSO
SPHINX31
P = 0.0055
P = 0.0145
F
P = 0.0002
P = 0.0003
G
P < 0.0001
H

Figure S8. Inhibition or knockdown of SRPK1 promotes endothelial ferroptosis, related to Figure 6

A. Intracellular ROS production (left) and semi-quantification (right) in endothelial cells of the indicated group. Scale bars, 100 µm.

B. EdU (red) and Hoechst (blue) staining in endothelial cells of the indicated group. The percentage of EdU-positive cells was quantified by ImageJ. Scale bars, 100 µm.

C. The levels of GSH in endothelial cells with knockdown of SRPK1 or corresponding control.

D. The levels of Fe^{2+} in endothelial cells with knockdown of SRPK1 or corresponding control.

E. Intracellular ROS production (left) and semi-quantification (right) in endothelial cells of the indicated group. Scale bars, 100 µm.

F. EdU (red) and Hoechst (blue) staining in endothelial cells of the indicated group. The percentage of EdU-positive cells was quantified by ImageJ. Scale bars, 100 µm.

G. The levels of GSH in endothelial cells treated with SPHINX31 or DMSO.

H. The levels of Fe^{2+} in endothelial cells treated with SPHINX31 or DMSO.

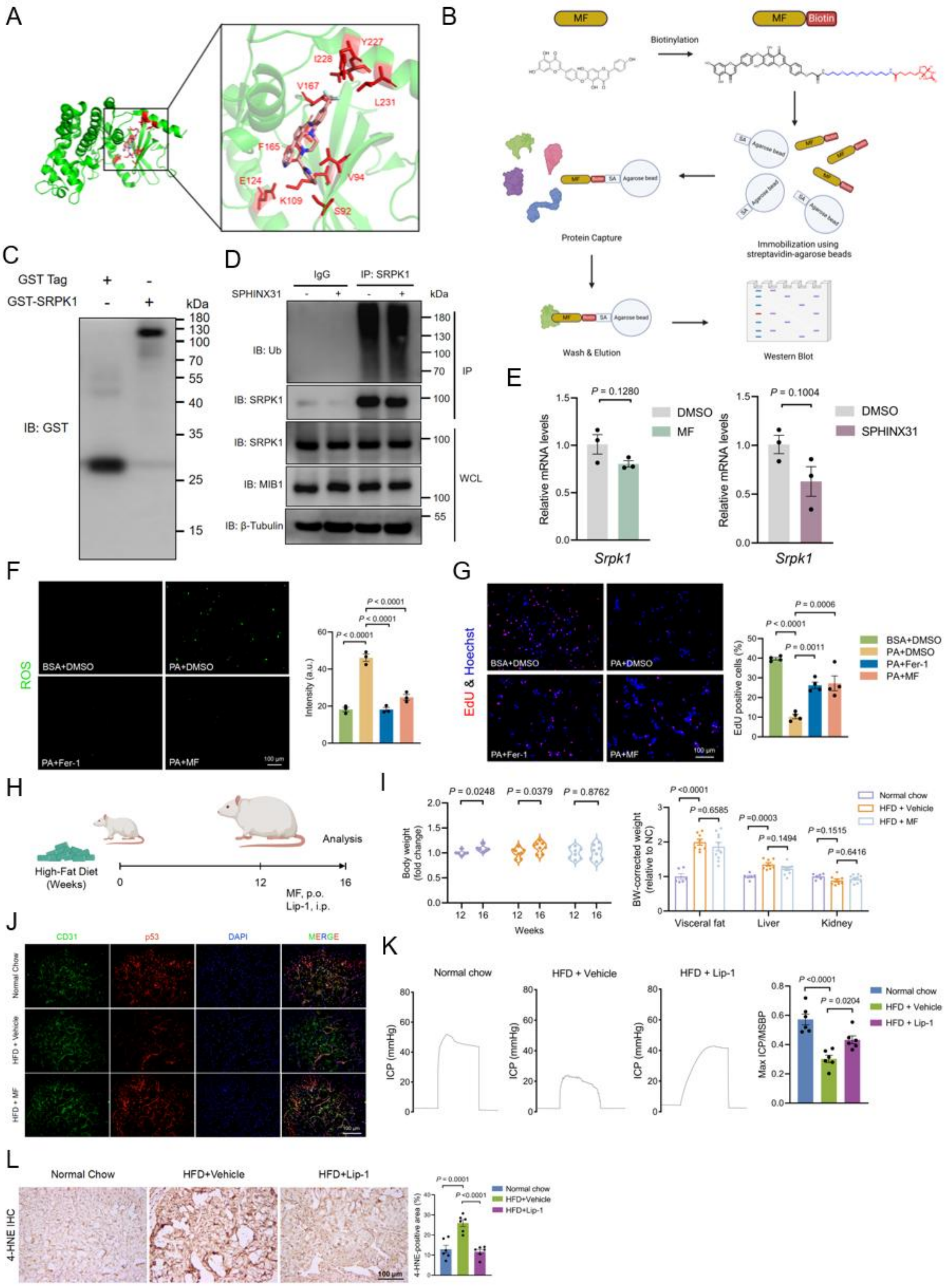

Figure S9. 4'-O-Methylochnaflavone interacts with SRPK1 and maintains its stability, related to Figure 7

A. The crystal structure (PDB ID: 5MY8) of SRPK1 (green) and its key residues (red) in complex with SPHINX31 (salmon).

B. Schematic figure of pull-down assays using streptavidin beads, biotin-MF, and endothelial cell lysates or recombinant proteins.

C. Immunoblot analysis and validation of recombinant GST Tag protein and GST-SRPK1 protein.

D. RCCEC were treated with SPHINX31 (5.0 μM, 24h) and MG-132 (10 μM, 6h) as indicated. Lysates were immunoprecipitated with anti-SRPK1, and immunoblot analysis was performed to analyze the levels of ubiquitination.

E. qPCR analysis of *Srpk1* mRNA in RCCEC treated with MF (1.0 μM) or SPHINX31 (5.0 μM) for 24h (n = 3 biological replicates).

F. Images of intracellular ROS production (left) and semi-quantification (right) in RCCEC in the indicated group. Scale bars, 100 μm.

G. EdU (red) and Hoechst (blue) staining in RCCEC in the indicated group. The percentage of EdU-positive cells was quantified by ImageJ. Scale bars, 100 μm.

H. Schematic diagram depicting HFD-fed animals that were administered with vehicle and indicated reagents for 4 weeks.

I. Body weight (BW) and BW-corrected weight of organs in male rats from the indicated group (n = 6-10 rats/group).

J. Immunofluorescence staining of corpus cavernosum tissues for p53 (red) and CD31 (green). Scale bars, 100 μm. Nuclei, DAPI, blue.

K. Representative intracavernous pressure (ICP) responses of male mice in the indicated group (n = 5-8 mice/group). The ratio of peak ICP/MSBP was calculated to evaluate the erectile function.

L. Immunohistochemistry images of 4-HNE in the corpus cavernosum of mice from the indicated groups (n = 5-8 mice/group). Scale bars, 100 μm.
